# From the inside out: Were the cuticular *Pseudonocardia* bacteria of fungus-farming ants originally domesticated as gut symbionts?

**DOI:** 10.1093/pnasnexus/pgae391

**Published:** 2024-10-15

**Authors:** Tabitha M Innocent, Panagiotis Sapountzis, Mariya Zhukova, Michael Poulsen, Morten Schiøtt, David R Nash, Jacobus J Boomsma

**Affiliations:** Centre for Social Evolution, Section for Ecology and Evolution, Department of Biology, University of Copenhagen, 2100 Copenhagen, Denmark; Centre for Social Evolution, Section for Ecology and Evolution, Department of Biology, University of Copenhagen, 2100 Copenhagen, Denmark; Medis 0454, INRAE, Centre INRAE Auvergne-Rhône-Alpes, Site de Theix 63122, France; Centre for Social Evolution, Section for Ecology and Evolution, Department of Biology, University of Copenhagen, 2100 Copenhagen, Denmark; Centre for Social Evolution, Section for Ecology and Evolution, Department of Biology, University of Copenhagen, 2100 Copenhagen, Denmark; Centre for Social Evolution, Section for Ecology and Evolution, Department of Biology, University of Copenhagen, 2100 Copenhagen, Denmark; Section for Protein Chemistry and Enzyme Technology, Department of Biotechnology and Biomedicine, Technical University of Denmark, 2800 Kongens Lyngby, Denmark; Centre for Social Evolution, Section for Ecology and Evolution, Department of Biology, University of Copenhagen, 2100 Copenhagen, Denmark; Centre for Social Evolution, Section for Ecology and Evolution, Department of Biology, University of Copenhagen, 2100 Copenhagen, Denmark

**Keywords:** mutualistic symbiosis, coevolution, attine ants, Actinobacteria, mutually exclusive microbiomes

## Abstract

The mutualistic interaction specificity between attine ants and antibiotic-producing Actinobacteria has been controversial because *Pseudonocardia* strains cannot always be isolated from worker cuticles across attine ant species, while other actinobacteria can apparently replace *Pseudonocardia* and also inhibit growth of *Escovopsis* mycopathogens. Here we report that across field samples of Panamanian species: (i) Cuticular *Pseudonocardia* were largely restricted to species in the crown of the attine phylogeny and their appearance likely coincided with the first attines colonizing Central/North America. (ii) The phylogenetically basal attines almost always had cuticular associations with other Actinobacteria than *Pseudonocardia*. (iii) The sub-cuticular glands nourishing cuticular bacteria appear to be homologous throughout the phylogeny, consistent with an ancient general attine-Actinobacteria association. (iv) The basal attine species investigated always had *Pseudonocardia* as gut symbionts while *Pseudonocardia* presence appeared mutually exclusive between cuticular and gut microbiomes. (v) Gut-associated *Pseudonocardia* were phylogenetically ancestral while cuticular symbionts formed a derived crown group within the *Pseudonocardia* phylogeny. We further show that laboratory colonies often secondarily acquire cuticular Actinobacteria that they do not associate with in the field, suggesting that many previous studies were uninformative for questions of co-adaptation in the wild. An exhaustive literature survey showed that published studies concur with our present results, provided that they analyzed field colonies and that Actinobacteria were specifically isolated from worker cuticles shortly after field collection. Our results offer several testable hypotheses for a better overall understanding of attine-*Pseudonocardia* interaction dynamics and putative coevolution throughout the Americas.

**Significance Statement**

Hosts and mutualistic symbionts co-evolve when interactions are specific enough to select for co-adaptation. The fungus-growing ants and their antibiotic-producing *Pseudonocardia* (Actinobacteria) are a case in point, but *Pseudonocardia* not being present or common on cuticles of many genera has caused controversy over coevolutionary explanations. However, many previous studies obtained actinobacterial sequences from lab colonies where secondary acquisitions are likely, or from extracts of entire ants rather than the cuticle alone, precluding separation of cuticular and gut microbiomes. We resolve these issues and suggest that there has been interaction specificity throughout the evolutionary history of ant fungus farming, but with shifting targets of co-adaptation because *Pseudonocardia* were initially gut symbionts and became cuticular symbionts late in attine evolution. Our data suggest that no other Actinobacteria have a history of co-adaptation with attine ants that is comparable to *Pseudonocardia*, but this needs to be validated by studies of south-American species.

## Introduction

Understanding the stability of mutualistic interactions over evolutionary time is a key challenge in evolutionary biology (1–4). Over the last three decades, the obligate farming symbiosis between attine ants and their fungal cultivars has become one of the best-studied model systems to address questions of this kind. These research efforts have realized major advances. We now have an exceptionally detailed understanding of the phylogenetic history of this ant tribe (5–9). It has also become clear that the symbiosis is more complex than originally appreciated, because it is challenged by specialized *Escovopsis* (Fungi: Hypocreaceae) mycopathogens (10–12). Farming ants have often, but not always, been found to rear Actinobacteria on their cuticle to control *Escovopsis* and possibly other diseases (13–17). Sequenced genomes have further corroborated earlier suggestions that dynamic antagonistic coevolution between cuticular *Pseudonocardia* and *Escovopsis* is likely for evolutionarily derived *Acromyrmex* leaf-cutting ants (18, 19). As a result, the number of mutualistic and parasitic partnerships in attine fungus farming has intrigued many as a possible example of advanced multipartite coevolution (15, 20–22). This idea has recently been reinforced by the identification of specific bacterial lineages associated with ant guts and adjoining organs, along with clarification of some of their complementary mutualistic services (23–26).

While the attine ants are clearly monophyletic (7, 27) as are most of their crop fungi ((28); only some *Apterostigma* spp. have subsequently switched to an unrelated coral fungus (29)), the coevolutionary interpretation of the interaction between attine ants, their cuticular Actinobacteria and *Escovopsis* infections in their fungus gardens has remained controversial. Workers of some attine lineages have been found to carry cuticular Actinobacteria other than *Pseudonocardia*, although active suppression of *Escovopsis* has rarely been demonstrated (30, 31). However, a detailed subsequent analysis has shown that the *Pseudonocardia* strains associated with attine ants in the field consist of six clades, among which signatures of co-cladogenesis with the ants are detectable even though some free-living strains are interspersed among the ant-associated strains (32). Two specific *Pseudonocardia* strains (*Ps1* and *Ps2*) can always be isolated from the cuticles of Panamanian leaf-cutting ants of the genus *Acromyrmex* (33). These strains correspond to clades IV and VI identified by Cafaro et al (32), which have now been genome sequenced and formally described as separate bacterial species (*Ps1*: *Pseudonocardia octospinosus*; *Ps2*: *Ps. echinatior*) (18, 34). We have previously shown (23) that a major change took place in the attine ant gut microbiome during the radiation of the tribe, including transitions to a markedly different gut bacterial community and from the presence to the absence of *Pseudonocardia* within the gut, that are perfectly correlated with ant phylogeny. The derived condition (absence of gut *Pseudonocardia*) is found in species that evolved in Central/North America and/or in contiguous Northeastern South America, north of the Andes (7, 35, 36), whereas the ancestral condition (presence of gut *Pseudonocardia*) is found in more basally diverging lineages that have a South American origin (7).

When evaluating the attine-*Escovopsis*-*Pseudonocardia* literature, it emerges that previous studies have used very different methods for isolating cuticular Actinobacteria (even within a single study), varying between simple washes of whole ants (e.g. 37–39), homogenizing ant worker bodies (e.g. 14, 30–32, 39), and dissecting tissues or scraping samples from specific cuticular regions (e.g. 34, 40–42). This implies that some studies likely reported bacterial communities from the cuticle only, while others could represent microbiota originating from inside ants. Studies of bacterial isolates require growth on media, which makes it unclear how prevalent they were in vivo (31, 40, 43). This variation in methods, combined with recent evidence that *Pseudonocardia* can consistently be isolated from dissected guts and associated organs of a number of phylogenetically basal attine ant species (23), prompted us to clarify the specific composition of the actively maintained cuticular microbiomes as they occur in field colonies of 11 Panamanian attine species.

Our objectives were to: (i) Evaluate the extent to which Actinobacteria in general and *Pseudonocardia* specifically are part of natural cuticular communities across attine species, (ii) Provide a cross-host-species comparison of the diversity and abundance of *Pseudonocardia* species on the cuticle and in the gut, including: (iii) Test whether cuticular Actinobacteria would show a similarly disjunct distribution between attine species that evolved before and after colonization of central/north America as we have documented for gut symbionts (23). (iv) Assess whether cuticular cultures of Actinobacteria are maintained by an evolutionarily homologous set of subcuticular glands no matter what cuticular structures later evolved (14), and (v) Review the literature to assess the extent to which combined extraction of bacterial DNA from cuticles and guts or secondary acquisitions of bacterial symbionts in the lab could explain most, if not all, of the controversy over the extent of coevolution between the multiple partners of the attine ant farming symbiosis.

To achieve these objectives, we used 16S rDNA amplicon sequencing of samples directly from field collected attine ants and exclusively from bacteria on the propleural plates, the almost invariably most biomass-dense patch of consistent actinobacterial growth in the attine species that maintain cuticular bacteria (14, 44). These analyses were combined with Transmission Electron Microscopy (TEM), a comparative review of previously published data on cuticular microbiomes, and an explicit comparison with earlier published data on gut microbiome composition across the same Panamanian attine species.

## Results

### The natural diversity of cuticular propleural-plate Actinobacteria in attine ant field colonies

**Fig. 1.**
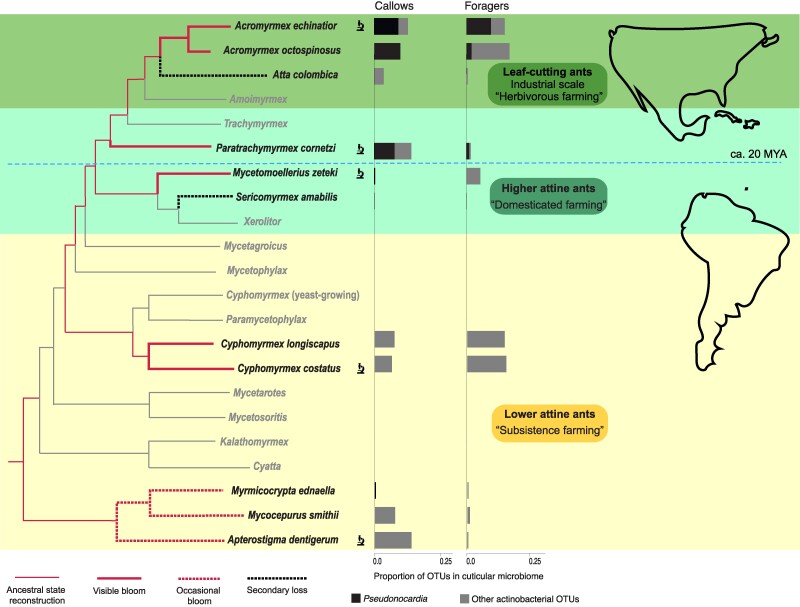
Simplified attine ant phylogeny for the genera occurring in the Panama canal zone (red branches) and genera with a South American or temperate North American distribution (gray branches) (modified from 6, 9, 27, 45), also highlighting the two main cultivar transitions after farming originated ca. 55–60 MYA: from “subsistence farming” of incompletely domesticated crops (lower attine ants; yellow shaded area) to specialized “domesticated farming” (higher attine ants; light green shaded area), and finally large-scale “herbivorous farming” in the leaf-cutting ants (darker green shading). Branches are marked to indicate which lineages (species) have visible white actinobacterial “blooms” on the propleural chest plates of at least some of the workers (major workers in *Acromyrmex*) in most colonies (solid red lines), or rarely and sparsely in only some colonies, and possibly under the forelegs in addition to, or instead of, the propleural plates (dashed red lines). The two black dashed branches represent secondary losses, with occasional very sparse presence of some white bloom on the head or thorax in *Sericomyrmex* and complete absence of visible bloom on the cuticle in *Atta* (13, 19, 44, 46). The bar charts show the mean proportion of actinobacterial OTUs relative to all bacterial OTUs identified from the cuticular propleural chest plate microbiomes of callow (newly hatched) and mature (foraging) workers on a scale from 0 to 25% (gray bars). The single 97% identity *Pseudonocardia* OTU identified from intestinal samples (*ActAcro1*; 23, 24) captured both *Ps1* and *Ps2* and essentially all other clades of attine-associated cuticular *Pseudonocardia* (black fractions of bars; see text and Figure S5, Tables S1 and S3 for details). The horizontal dashed blue line marks the inferred colonization of Central and North America ca. 20 MYA by the common ancestor of *Paratrachymyrmex*, *Trachymyrmex* and the leaf-cutting ants, well before complete land-bridge closure of the Panama Isthmus (7). Those species used for TEM (Figure 2) are marked with a microscope symbol.

We obtained rarefied 16S rDNA community profiles for 194 individual propleural plate samples (62 colonies; 11 species), which yielded a total of 247 OTUs, of which 30 were actinobacterial (Table S1a). OTU richness varied significantly among the 11 ant species (GLMM, rarefied dataset; *Wald χ²* = 96.96, d.f. = 10, *P* < 0.0001), with the most abundant OTUs (001–003) being ubiquitous soil bacteria present across most samples, independent of species (Tables S1 and S2). In general, OTU richness on individual workers (range 1–33), and within colonies (range 2–54) was relatively low compared to the total number of OTUs (247). Only three of the 11 attine species had abundant *Pseudonocardia,* primarily on newly eclosed (callow) workers, but also on foragers (Figure 1; Table S3). Two of these were *Acromyrmex* leaf-cutting ants, consistent with earlier findings (34), and the third was a higher nonleaf-cutting attine ant, *Paratrachymyrmex cornetzi* (5, 6). The phylogenetically more basal higher attine *Mycetomoellerius zeteki* (6) had very different actinobacterial communities. As expected (14, 44), *Atta colombica* leaf-cutting ants and *Sericomyrmex amabilis* (a “higher” nonleaf-cutting attine ant) did not have Actinobacteria on their propleural plates, consistent with lack of visible white blooms (Figure 1), and with these ants exclusively using hygienic behaviors and metapleural gland secretions to control *Escovopsis* (46–48).

The five species of phylogenetically basal “lower” attines had cuticular microbiomes containing Actinobacteria other than *Pseudonocardia* (e.g. *Corynebacterium, Kineosporia, Nocardia*) but without any OTU being consistently present in any species (Table S1 and S3b). However, some cuticular *Pseudonocardia* were found on callow workers of *Apterostigma dentigerum* and in traces on *Cyphomyrmex costatus*. These patterns did not fundamentally change in older foraging workers except that rare *Pseudonocardia* tended to become more abundant, and prevalent *Pseudonocardia* less abundant in foragers (Table S3). All but one *Pseudonocardia* sequence that we retrieved were part of a single 97% similarity OTU (OTU004) which was identical to *ActAcro1* (Table S1), previously identified as a common gut symbiont OTU of the lower attine ants included in our present study (23; see also below). The single exceptional sequence (OTU0105) was 96% similar to *ActAcro1*, suggesting that all attine ant-associated *Pseudonocardia* OTUs belonged to a single, genetically homogeneous lineage. This pattern of significant differences among ant species in actinobacterial and *Pseudonocardia* OTUs was essentially the same regardless of whether rarefied (Actinobacteria: *Wald χ²* = 27.6, d.f. = 10, *P* = 0.016) or unrarefied (Actinobacteria: *Wald χ²* = 28.5, d.f. = 10, *P* = 0.0015) richness (Table S2), rarefied (Actinobacteria, *Wald χ²* = 41.5, d.f. = 10, *P* < 0.0001) or unrarefied (*Pseudonocardia*, *Wald χ²* = 340.40, d.f. = 10, *P* < 0.0001) abundance (Table S4), or prevalence (Actinobacteria, *Wald χ²* = 70.74, d.f. = 10, *P* < 0.0001, *Pseudonocardia*, *Wald χ²* = 30.93, d.f. = 10, *P* = 0.0006 ; Table S5) were considered.

Although we do not know whether OTUs other than *Pseudonocardia* have functional roles, our analyses suggest that the prevalence (proportional representation) of actinobacteria and the overall composition of actinobacterial cuticular microbiomes (*Pseudonocardia* vs other actinobacteria) differs across the attine clade, both when comparing the presence/absence of visible bacterial blooms on the propleural plates (GLMM, unrarefied dataset, proportion actinobacteria; *Wald χ²* = 17.49, d.f. = 2, *P* = 0.0002; proportion *Pseudonocardia*; *Wald χ²* = 11.6, d.f. = 2, *P* = 0.0030; Table S5) and when contrasting the basally diverging lineages that evolved in South America with the crown group inferred to have evolved in or adjacent to Central/North America (7, 35) (GLMM, unrarefied dataset, proportion actinobacteria; *Wald χ²* = 6.22, d.f. = 1, *P* = 0.0126; proportion *Pseudonocardia*; *Wald χ²* = 7.35, d.f. = 1, *P* = 0.0067; Figure 1; Tables S4 and S5). Thus, while actinobacterial species richness varied across the Panamanian attine ants that we sampled, the presence/absence of *Pseudonocardia* appears to be consistent (Table S3), as was previously documented for the gut symbionts of the same attine species (23).

The 30 actinobacterial OTUs retrieved from our total sample of propleural plates included 28 other potential antibiotic producers (Tables S1 and S3), but we did not find actinobacterial genera previously reported from the cuticles of attine ants maintained in the lab, such as *Streptomyces* or *Amycolatopsis* (31, 34, 37, 49, 50), either in addition to, or instead of, *Pseudonocardia*. Given our extensive field sampling, this strongly suggests that attine ants maintained in captivity are prone to secondary acquisitions of Actinobacteria that do not occur in their natural habitats and thus cannot have specific co-adapted functions. Consistent with this finding, we found *Pseudonocardia* on the cuticles of *M. zeteki* workers collected for microscopy from colonies maintained in the lab for 6–18 months, even though ants of this species did not have cuticular *Pseudonocardia* when sampled in the field. Some other *M. zeteki* workers in our microscopy samples had acquired *Amycolatopsis* rather than *Pseudonocardia* (Table S6), an association that has been reported for lab colonies of this species before (31, 34), but does not appear to occur naturally either. Our MiSeq data therefore indicate that only field samples are informative about natural associations between attine ants and cuticular Actinobacteria.

### Early attine ants acquired Actinobacteria, not *Pseudonocardia*, on their cuticles

**Fig. 2.**
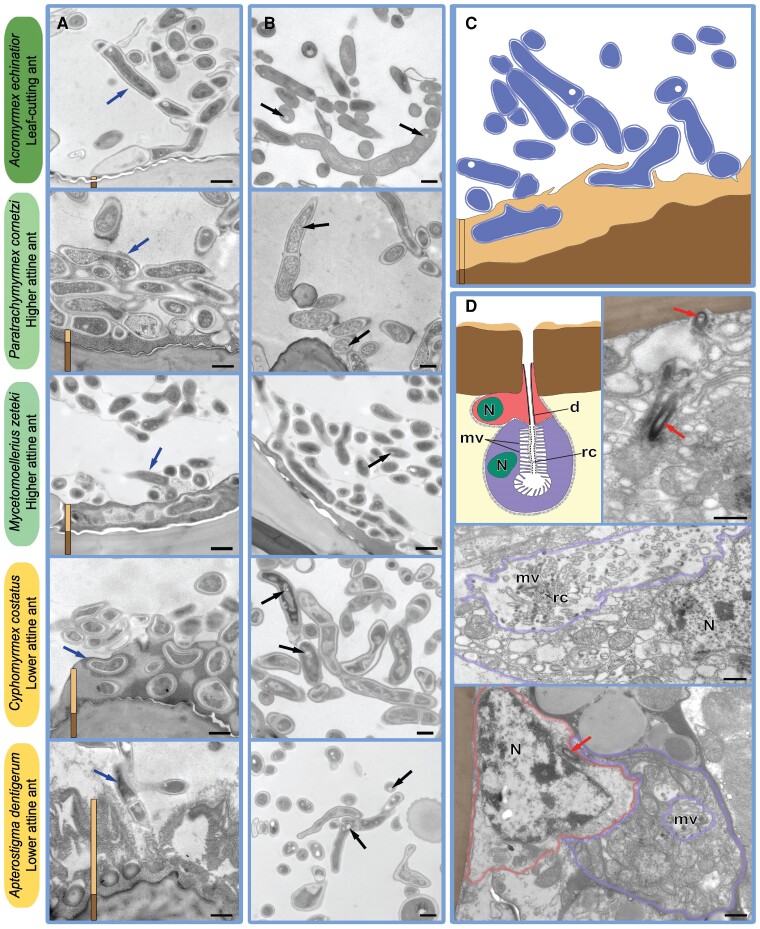
Key morphological characteristics (TEM) of cuticular bacterial ectosymbiont growth across five Panamanian species of attine fungus-growing ants, all located on the propleural plates (or the mesothorax in *Ap. dentigerum*) and spanning the phylogenetic diversity of the attine ants (Figure 1). Column A: The adherence interface between bacteria and cuticular structures. Column B: The typical filamentous habitus of cuticular Actinobacteria. Panel C: Schematic representation of typical filamentous bacteria (blue) with light (white circle) inclusions in their cytoplasm, growing on a layer of secreted substance (sandy yellow) directly attached to the ant cuticle (brown). These structures are also highlighted in column A with vertical bars in the same colors and blue arrows pointing towards filamentous bacteria, and in column B where black arrows point to light inclusions within bacterial cells. Panel D: Schematic diagram and TEM images of a typical bicellular exocrine gland directly below the propleural plates of the higher attine, nonleaf-cutting ant *P. cornetzi*; these glands produce and secrete unknown nutritional resources for maintaining *Pseudonocardia*-dominated microbiomes (see Figures S1–S3 for images and descriptions of analogous glands in the four other species investigated, and Table S6 for accompanying sequencing data). The glands are comprised of a duct cell (red) and a secretory cell (violet), situated immediately below the cuticle (brown), where they are spaced out with mean distances of ca. 20 µm in the central areas of the propleural plates (see Figure S4 for details). Abbreviations denote cell nuclei (**N**), microvilli (**mv**), the receiving canals (**rc**), and ducts (**d**). Ducts are highlighted with red arrows in the adjacent TEM images, and cell walls for the two cell types are drawn in the images using the same colors as in the schematic diagrams. Scale bars are 0.5 µm.

We used TEM to visualize the structure of the subcuticular nourishment glands of the propleural plate that are connected by narrow ducts to the surface where Actinobacteria grow (14, 44) for five attine species from across the phylogeny (Figure 1) that had been maintained in the laboratory. The high resolution of TEM enabled us to identify a layer of substrate for actinobacterial growth on the cuticular surface, and to obtain morphological evidence of exocrine glandular secretion (Figure 2, Figures S1–S3). In all five attine species, the subcuticular glands were composed of a large secretory cell and an accompanying duct cell (or two such cells) that carries secretion to the exterior (Figure 2D and Figures S1–S3). The structure of the gland-cuticle-complex appears to be somewhat less elaborate in basal *Apterostigma* (Figure S3) compared to *Cyphomyrmex* and *Paratrachymyrmex* (Figure 2 and Figure S2), and to have additional evolutionarily derived characteristics in *Acromyrmex* species (Figure S1). The combined information thus suggests that it is most parsimonious to infer that these subcuticular glands are homologous, i.e. that they have a single evolutionary origin and became elaborated later on without a fundamental change in function in contrast to the externally visible cuticular crypts and tubercles that underwent various elaborations and reductions (14).

The combined images are consistent with the substances produced by the subcuticular glands mediating bacterial nutrition and potentially supporting attachment of Actinobacteria to the cuticle (Figure 2; Figure S4). The observed filamentous bacterial morphology on propleural plates of workers of all five lab-maintained species is typical for Actinobacteria. The images thus corroborated the OTU data showing the consistent cuticular presence of Actinobacteria particularly in lab workers, but also in field workers where proportional abundances of actinobacterial OTUs normally remained below 25% (Figure 1; Tables S6 and S7). Most of our TEM images showed electron translucent inclusions in the actinobacterial cytoplasm (black arrows in Figure 2B and Figure S3), which might be gas vesicles associated with pathways for regulating secondary metabolite synthesis, as has been suggested for other Actinobacteria (51). The TEM work thus suggests that attine ants evolved structural and functional adaptations for hosting cuticular Actinobacteria shortly after the origin of fungus farming, but that further inferences to suggest that these early cuticular communities must have involved *Pseudonocardia* are unwarranted.

### Cuticular and gut *Pseudonocardia* appear to be mutually exclusive

**Fig. 3.**
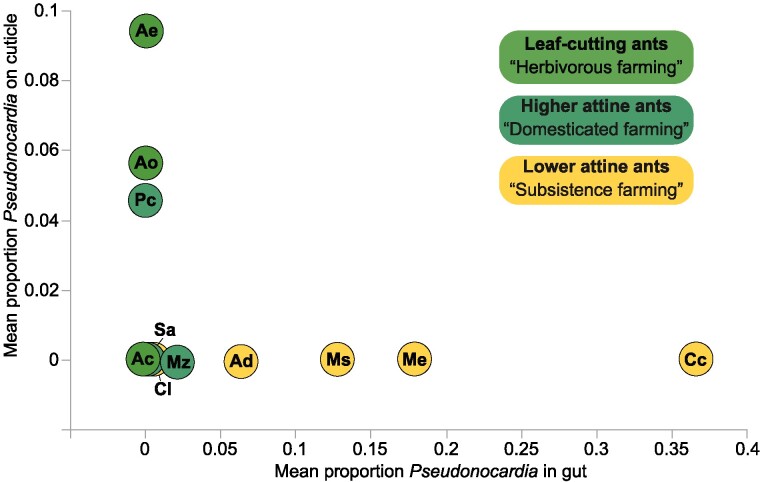
Mean proportions of cuticular propleural plate *Pseudonocardia* sequences (relative to all bacterial sequences; Figure 1; Table S3) *versus* mean proportions of *Pseudonocardia* sequences from homogenized (Me, Ms, Cc, Cl) or dissected (all other species) ant gasters (relative to all bacterial sequences; data from (23); see Table S8). The comparison focused on the single *Pseudonocardia* OTU (*ActAcro1*; 23, 24) that was unambiguously identifiable in both datasets using a 97% similarity threshold. This OTU contained representative sequences for the two known cuticular *Acromyrmex* leaf-cutting ant symbiont species (*Ps1* or *Pseudonocardia octospinosus* and *Ps2* or *Pseudonocardia echinatior* (18)), and for 85 of the 87 published attine-associated *Pseudonocardia* sequences isolated from host ant genera spanning the attine phylogeny by Cafaro et al (32); the two exceptions were a *M. zeteki* and an *Ap. dentigerum* sample of *Pseudonocardia* that were both >96% similar; details in Table S9. Attine species codes: Ac, *Atta colombica*; Ae, *Acromyrmex echinatior*; Ao, *Acromyrmex octospinosus*; Pc, *Paratrachymyrmex cornetzi*; Mz, *Mycetomoellerius zeteki*; Sa, *Sericomyrmex amabilis*; Cc, *Cyphomyrmex costatus*; Cl, *Cyphomyrmex longiscapus*; Ad, *Apterostigma dentigerum*; Ms, *Mycocepurus smithii*; Me, *Myrmicocrypta ednaella*.

In a recent study, Sapountzis et al (23) showed that *Pseudonocardia* OTUs are consistently present inside the gasters (fourth to last abdominal segments) of field sampled lower attine ants in Panama, that they are only variably present in the gaster organs of similar samples of higher, non-leaf-cutting attines, and absent as symbionts in guts and associated organs of leaf-cutting ants. The single 97% OTU (*ActAcro1*) identified in that and a previous study (24) captured almost all of the sequence diversity for cuticular *Pseudonocardia* in our present dataset (see above), including the genome sequenced *Ps1* and *Ps2* cuticular ectosymbiont species of *Acromyrmex* leaf-cutting ants (18, 32, 34). This match allowed us to directly compare our new amplicon sequence data from the propleural plates with those obtained for the gut microbiomes in the same set of attine ant species from the same Panamanian site (23). This revealed a surprisingly consistent contrast between the two microbiomes (Figure 3; Table S8) with almost none of the colonies across the 11 investigated species having *Pseudonocardia* both in the guts and on the cuticles of workers. The two lineages that have secondarily lost cuticular Actinobacteria (represented by *At. colombica* and *S. amabilis* (46)) did not have any *Pseudonocardia* in their guts either (Figure 1; Table S8).

The pattern in Figure 3 implies that whenever a wild Panamanian attine ant has a clear association with *Pseudonocardia* (i.e. many *ActAcro1* OTU sequence hits relative to any other actinobacterial OTU hits), these bacteria will occur either inside the body or on the cuticle, but not both (see also Table S8). Comparing our results with the six clades of attine-associated *Pseudonocardia* that were phylogenetically mapped by Cafaro et al (32), it emerged that the three cuticular microbiomes with substantial *Pseudonocardia* presence were always *Ps1* (*Ps. octospinosus*) or *Ps2* (*Ps. echinatior*), i.e. Clades IV and VI of Cafaro et al (32), respectively, in *Acromyrmex* species, and *Ps1* or Clade V when associated with *P. cornetzi* or the lower attine *Ap. dentigerum.* The remaining three *Pseudonocardia* clades identified by Cafaro et al (I, II, and III) were basal in the overall *Pseudonocardia* tree, and associated with gut (but not cuticular) microbiomes of lower attine ants and *M. zeteki* (Figure S5). We further compared the available 16S rDNA sequences of Cafaro et al (32) with the representative *ActAcro1* sequence of the joint gut and cuticular microbiomes (generated from all 16S sequences encompassed by the 97% identity OTU in this study and 23). This showed that 85 of the 87 combined sequences of attine-ant-associated *Pseudonocardia* strains analyzed by Cafaro et al (26) (covering all 6 clades) had >97% similarity with *ActAcro1* (the two exceptions were >96% similar; Figure 3; Tables S1b, S9).

Our comparisons of the 404 amplicon sequences drawn from the cuticular samples (this study) and the 1,095 amplicon sequences from the gut dataset (23), all within the *ActAcro1* 97% OTU, showed a high degree of similarity, with only minor differences between the four most abundant gut-derived sequences and the five most abundant cuticular-derived sequences (Table S10). This illustrates that these *Pseudonocardia* communities are very similar in spite of a few outliers (e.g. the Gut1 sequence in Table S10). Comparison of all other OTUs obtained from inside gasters across the attine host species (23) further revealed that a number of them seem to be favored or repressed depending on whether *ActAcro1* is present or not in the cuticular microbiome (Table S11). This finding complements the results obtained by Sapountzis et al (23), which showed that the presence/absence of unspecified cuticular actinobacterial blooms (rather than the confirmed presence of cuticular *ActAcro1* as we document here) was positively or negatively associated with the overall composition of gut microbiomes.

### A controversy driven by disparate sampling?

**Fig. 4.**
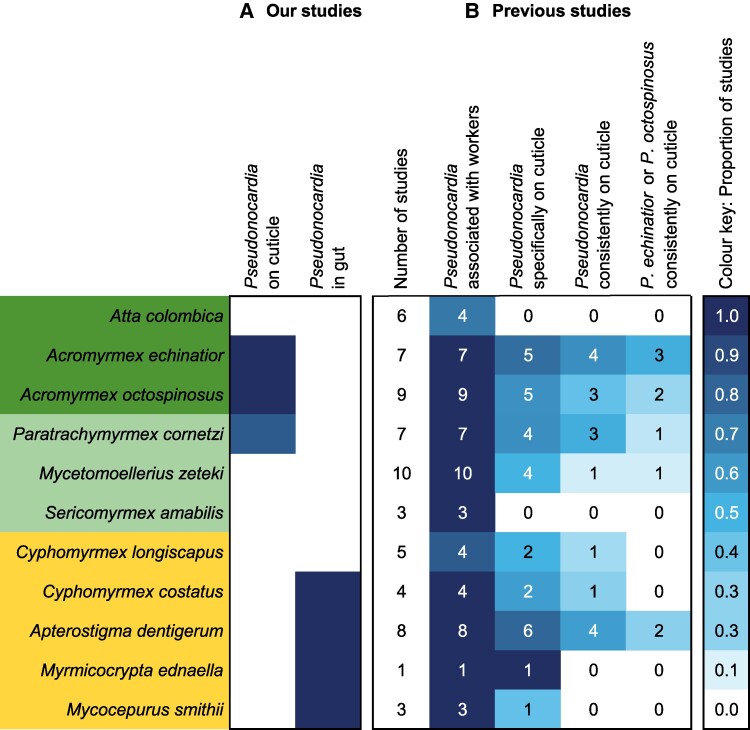
Comparison of our present results with previously published studies: (A) the mean proportion of *Pseudonocardia* sequences on the cuticle and within gasters (guts and associated organs) across 11 Panamanian attine species: left column, this study; right column, data from Sapountzis et al (23). (B) The proportion of previous (pre 2019) studies reporting associations of *Pseudonocardia* with attine ants but without explicitly considering whether they could be gut or cuticular symbionts, broken down by four increasingly stringent likelihood criteria for correctly assigning sequences as truly being part of cuticular microbiomes. Shades of blue reflect the degree of confirmation of *Pseudonocardia* sequences across colony-level samples in our present study (A) and across previous studies (B) with numbers within the four columns of (B) referring to the number of studies that actually sampled ant workers. A few studies that sampled other ant castes or fungus garden material were not directly comparable and therefore not included here (see Table S12 for details on the criteria used for the four categories of sampling stringency in (B), and Figure S5 for comparison to *Pseudonocardia* clades I–VI in Cafaro et al. (32)).

Finding that *Pseudonocardia* on the cuticle and in the gut belong to a single *ActAcro1* OTU with a consistent, mutually exclusive presence prompted us to review the entire literature that had made *Pseudonocardia* associations across attine ants controversial (pre 2019; Table S12). We found that essentially all taxonomic ambiguities could be explained as secondary lab acquisitions and that the literature data converged on our present results when we only considered field samples taken from worker cuticles that were investigated with the same stringent sampling criteria as in the present study (Figure 4).

## Discussion

Our results confirm that there is a long-term association between attine ants and *Pseudonocardia*, as asserted by Cafaro et al (32). However, our data imply that *Pseudonocardia* were initially gut symbionts, that only a later crown group of *Pseudonocardia* (at least for its Panama canal-zone representatives) secondarily evolved to be cuticular ectosymbionts, and that establishment of *Pseudonocardia* on the propleural plates coincided with their disappearance as gut symbionts. A recent biogeographic analysis (7) indicates that this transition most likely happened when the first representatives of attine ants colonized Central and North America ca. 20 MYA, possibly during a former land–bridge connection (52). In contrast, and as far as Panamanian species are concerned, the more basally diverging attine ant lineages that evolved earlier in South America (7), retained their ancestral cuticular bacteria, which appear to only very rarely contain *Pseudonocardia*. Our TEM microscopy images showed that the subcuticular glands that provision the attine cuticular bacteria (44) have a highly conserved morphological structure with only minor modifications, no matter which Actinobacteria they provision. This contrasts with the nonconserved secondary elaborations and reductions of the externally visible cuticular crypts and/or tubercles that affect how the secreted glandular substance (Figure 2C, D) is distributed on the cuticular surface as recently documented by Li et al (14). This interpretation is consistent with secondary losses of these glands and the actinobacterial cultures depending on them always having occurred in terminal lineages and not at deeper nodes (14, 44).

The recent studies by Branstetter et al (7) and Barrera et al (35) fundamentally changed the way we understand the evolutionary history of the attine fungus-farming symbiosis because they showed that the crown group lineages, including *Amoimyrmex*, *Atta*, and *Acromyrmex* leaf-cutting ants, *Trachymyrmex*, and *Paratrachymyrmex* evolved in or directly adjacent to Central/North America and colonized South America only after the Isthmus had reconnected 5–10 MYA (52). Recent microbiome work has confirmed that this major vicariance event coincided with a significant transition in diversity and density of gut microbiota (23) and with the acquisition of novel gut symbionts with specific mutualistic functions (25). Our combined results indicate that the genus *Mycetomoellerius*, represented by Panamanian *M. zeteki* in our studies, likely retained an ancestral, possibly South American (7) microbiome, and that the ancestor of the genus *Paratrachymyrmex* (represented by Panamanian *P. cornetzi* in our studies) and the two leaf-cutting ant genera represent the crown group that flipped towards a dual set of evolutionarily-derived microbiomes (Table S3). In this evolutionarily derived attine clade, the cuticular microbiome could be secondarily lost in a single species adapted to desert habitat (*Acromyrmex versicolor*) and in entire genera like *Atta* (14). Our results confirm that *Ap. dentigerum* is special in being the only basal attine ant that has crown group *Pseudonocardia* strains in its cuticular microbiome on both callows and foragers, albeit at very low levels and inconsistently across colonies. This adds to earlier observations highlighting *Apterostigma* as an exceptional genus of phylogenetically basal fungus-farming ants: it is the only attine lineage with a subclade that secondarily recruited an unrelated cultivar that ancestrally decomposed wood rather than leaf litter (Pterulaceae; see 28, 53), and it has secondarily acquired an evolutionarily derived leaf-cutting ant cultivar in at least one species (*Apterostigma megacephala*; 54).

The study by Barrera et al (35) essentially only differed from Branstetter et al (7) by suggesting that the *M. zeteki* lineage also evolved in subcontinental vicariance-isolation rather than in smaller-scale isolation (rainforest vs drier habitat) in central South America, as inferred by Branstetter et al (7) based on a substantial sampling effort across the crucial genera around the origin of the higher attine ants. In this context, it is important to note that the study by Barrera et al (35) was focused on the genus *Atta* so that taxon-sampling was not as well-suited as the coverage by Branstetter et al (7) to address the timing of the isolation of higher attine ants from South America. It is for this reason that we have used the Branstetter et al (7) inferences for interpreting our results. In case future work would show that the other interpretation (35) would prevail, the implication would be that the microbiome transition between the genera *Mycetomoellerius* and *Paratrachymyrmex* would possibly require a less parsimonious two-step explanation that would not be linked to the climate change argument by Branstetter et al that the emergence of the higher attine ants was associated with a reduction in rainforest habitat and a substantial increase in dry grassland habitat within South America.

We found strong support for previous observations (30, 34, 37, 40, 55) and theoretical predictions (56) that cuticular microbiomes of all attine ants with subcuticular glands to nourish them are sensitive to invasion by other antibiotic-producing bacteria. However, this invasibility only appears to have led to Actinobacteria other than *Pseudonocardia* becoming abundant in long-term laboratory colonies. It is therefore significant that we could not retrieve any *Streptomyces* as natural cuticular symbionts of *Acromyrmex* field colonies, nor any *Amycolatopsis* as natural cuticular symbionts of *M. zeteki* field colonies. This is consistent with many literature records of these other actinobacterial genera being based on samples from ants reared in the lab rather than on sampled cuticles of field-collected ants (e.g. 30, 31, 34; see Table S12 for further details). Although some of our categorical assignments (Figure 4) may not be completely certain (Table S12), none of the possibly ambiguous cases offer hard evidence against our inference that *Streptomyces* and *Amycolatopsis* are rarely if ever naturally occurring on the cuticles of, respectively, *Acromyrmex* and *Mycetomoellerius* species in Panama. The results in Figure 4 imply that field sampling is crucial for investigating signatures of possible co-evolution, while the mutual exclusiveness pattern in Figure 3 indicates that separate sampling of guts and cuticles is necessary to infer where possible co-adaptation might have occurred. Differential sampling of gut and cuticular microbiomes has not been standard practice and this is bound to have produced inaccurate interpretations. For example, similar to earlier work, a recent comparative study by Li et al (14) homogenized sets of entire worker ants to verify associations with *Pseudonocardia* and inferred that these bacteria must have come from the cuticle without specifically checking cuticular and gut microbiomes. Our results (Figure 4) also underline that culture-dependent methods for amplifying Actinobacteria before sequencing are unlikely to reflect natural representations of bacterial diversity in cuticular microbiomes of attine ants.

The history of the decade-long disagreement between research groups about the attine-*Pseudonocardia* interaction-specificity is intriguing. The first studies inferred that all white cuticular blooms across the phylogenetic branches of the attine ants must have *Pseudonocardia* as key components and that these actinobacterial symbionts must be exclusively vertically transmitted (13, 15, 20, 57). When this first inference did not hold up, it was argued that horizontal acquisition must be a dominant force (30, 31) and later studies appeared to support a mixture of vertical and horizontal acquisition. Actinobacteria other than *Pseudonocardia* were found to be dominant on lab-reared attine ant workers (34, 37, 39), albeit with Haeder et al (49) appearing to be the only study to make such a mixed transmission inference based on ants that were recently collected from the field. Another amplicon sequencing study showed that bacteria on *Acromyrmex* cuticles became more diverse as workers aged and started foraging outside the nest (34). Our present mutual exclusiveness result of *Pseudonocardia* in cuticular and gut microbiomes (Figure 3) suggests that the original vertical acquisition hypothesis (13, 15, 20) is correct, but that this assertion remains to be proven by dissection and sequencing of the guts and associated organs of dispersing gynes in the phylogenetically basal attine lineages that evolved in South America. Our present results also suggest that there may be other vertically transmitted genera of Actinobacteria on the cuticles of *Cyphomyrmex* species, some *Apterostigma*, and the clade to which *M. zeteki* belongs (14), but that none of these is likely to have a consistent history of co-adaptation with attine ants comparable to *Pseudonocardia*.

Against this background, it appears that much if not all of the controversy over the extent of coadaptation between attine ants and *Pseudonocardia* symbionts (10, 13, 30–32, 34, 37) is due to: (i) Previous research having ground up whole ants, rather than sampling abdominal (gaster) and thoracic (propleural plate) microbiomes separately (e.g. 31, 39; see Table S12 for further details), (ii) Interpreting lab-based associations with Actinobacteria as necessarily being representative of natural associations (e.g. 30, 31), and (iii) Considering incidental or recurrent horizontal acquisition of new actinobacterial OTUs by lab-reared attine ants as evidence for lack of co-adaptation with *Pseudonocardia* (30). As a previous model based on screening theory argued (56), some horizontal acquisition dynamics are likely to be a natural part of keeping cuticular bacterial communities healthy, as long as there is a single native symbiont that monopolizes newly hatched workers, a contention consistent with recent evidence (58). These workers will then in turn inoculate the cuticular microbiomes of their dispersing gyne (future queen) siblings so that faithful vertical transmission of the native symbiont is secured (34, 59). Secondary acquisitions of other bacteria on older workers are unlikely to compromise this course of events because these workers do not have nursing tasks. This is consistent with the native actinobacterial symbionts *Ps. octospinosus* (*Ps1*) and *Ps. echinatior* (*Ps2*) having been conserved within generic ant lineages (i.e. across *Acromyrmex* species) and between higher attine genera (i.e. the Panamanian *Paratrachymyrmex* and *Acromyrmex* species) and not being replaceable by horizontal transmission (34, 59).

In light of the findings by Branstetter et al (7) and Barrera et al (35) that at least a subset of the higher attine ants arose in Central and North America, it would be interesting to reconstruct the native actinobacterial symbiont(s) of the common ancestor of extant *P. cornetzi* and *M. zeteki* in Panama. The former has attine crown-group *Pseudonocardia* as native cuticular symbionts (*P. octospinosus* and a Cafaro, et al (32) symbiont from Clade V; Figure S5), very different from *M. zeteki* for which it remained unclear whether or not it has a single native cuticular symbiont. Our present results are consistent with irreversible changes in the association between attine ants and co-adapting *Pseudonocardia* having been rare after *Pseudonocardia* appeared in cuticular microbiomes. This would predict that the extant representatives of the crown group attine ants may have retained microbiome signatures that basal genera that evolved in South America lack. This implies that we would expect that the *Paratrachymyrmex* and *Acromyrmex* species that later colonized South America have continued to carry these signatures. We would even add the stronger prediction that both North American *Trachymyrmex septentrionalis* and the recently described sister genus of the leaf-cutting ants, *Amoimyrmex* (9) are also expected to have retained (or secondarily lost) *Pseudonocardia* dominated cuticular microbiomes and gut microbiomes without *Pseudonocardia*, in spite of the latter currently having an exclusive South American distribution.

Detailed investigations of the possible adaptive significance of *Pseudonocardia* in the guts of basal attine ants, including the higher attine *M. zeteki* branch, were beyond the scope of our present study but should be a priority for future work. The original inference (13) that *Pseudonocardia* coevolved with the attine ants on their worker cuticles throughout the tribe's evolutionary history of >50 MY now appears to be incorrect, but that does not imply that consistent domestication of *Pseudonocardia* as a biocontrol agent against *Escovopsis* is an untenable hypothesis (32). *Pseudonocardia* is a relatively uncommon actinobacterial genus that is rarely isolated from soil samples (60), so its consistent and relatively abundant presence in the guts of phylogenetically basal Panamanian attine ants (23) would be surprising if these bacteria had no functional role and were not vertically transmitted as well. If gut *Pseudonocardia* provides the fecal droplets of attine ants—normally used to transfer fungal and ant enzymes within fungus-gardens (61, 62)—with properties that inhibit *Escovopsis* growth, it would not seem far-fetched to imagine that *Pseudonocardia* domestication started independently of the subcuticular glands that allowed phylogenetically basal attine ants to horizontally acquire other Actinobacteria for complementary hygienic purposes. This inference would predict that hygienic behaviors related to fecal droplet deposition should be different between *P. cornetzi* and *M. zeteki*, and that these sympatric Panamanian species might have specialized strains of *Escovopsis* that are largely ineffective against fungus gardens of the other species. Provisional evidence suggests this may indeed be the case (63).

As our study remained restricted to Panama canal-zone species, further work will be required to unravel the origin of *Pseudonocardia* associations with the guts and cuticles of attine ants throughout their American ranges. It recently became clear that the sister lineage of the entire clade of fungus-growing ants, the specialized predatory Dacetine ants (7), have no associations with *Pseudonocardia* (14). This suggests that obligate fungus farming (7), the emergence of the genus *Escovopsis* (11, 64), the acquisition of *Pseudonocardia* gut symbionts (23, 24), and the cultivation of non*Pseudonocardia* bacteria on the worker cuticule (this study) followed each other in rather quick succession during early attine ant evolution some 55–60 MYA (14, 65). Whether the exclusive fungal diet of ancestral attine ants facilitated colonization of the gut microbiome by *Pseudonocardia* remains unknown, but this major shift to a uniform and easily digestible diet may have implied that aggressive digestive enzymes disappeared (66) and that gut pH changed, as reported for other significant diet shifts (67). Clarifying which genomic differences are responsible for gut *Pseudonocardia* to live internally and anaerobically and cuticular *Pseudonocardia* to live externally (aerobically) should also be a priority, similar to answering why putative *Escovopsis* control by *Pseudonocardia* in attine ant guts became obsolete after they colonized Central and North America.

## Materials and methods

We collected both newly eclosed (callow) and older foraging workers from field colonies of 11 attine species (226 individuals across 62 colonies; details in Table S1) in Soberania National Park, Panama, and stored them in 99% ethanol. We dissected the propleural plates of each individual worker under sterile bench conditions at the University of Copenhagen immediately upon return (giving a time to dissection of approximately 3–6 weeks post collection, but with protection from exposure to secondary contamination by keeping the newly collected colonies in a separate rearing room). In one species (*Ap. dentigerum*) cuticular blooms may be more visible on other parts of the worker body (13, 14), but we chose to standardize bacterial sampling throughout all ant species rather than introducing an inconsistency that would have had the same implicit assumption that cuticular communities have comparable bacterial species composition across cuticular segments of the same individual ant. We then extracted the DNA using the Qiagen Blood & Tissue DNA kit and following the manufacturers’ specified protocol, with the additional step of vortexing each sample for 30 seconds with 0.1 mm glass beads at the first stage of DNA extraction; three blank DNA extractions (i.e. simultaneously using the same extraction protocols and extraction reagents on water samples) were included as negative controls. Having identified optimal library preparation conditions and confirmed the negative controls using 16S rDNA PCR reactions, we sent all DNA samples to the Microbial Systems Laboratory at the University of Michigan for library preparation and Illumina MiSeq sequencing (protocol described in Caporaso et al (68); Supplementary material).

Raw sequence data were analyzed in *mothur* (v 1.36.1; mothur.org page first accessed 2015 July 27) using the standard operating procedure developed for Illumina MiSeq data with modifications as described in Kozich et al (69). Sequences were aligned and classified using the SILVA 111 nonredundant database (70). Operational taxonomic units (OTUs) were identified using 97% similarity, and classifications were assigned using the grouped sequences (Supplementary material). We rarefied data at a threshold of 2,000 reads and examined for under-sampling, removing 32 of the 226 individual samples. This reduced the final number of OTUs from 508 to the 247 used for subsequent analyses (unless specified otherwise). In addition to examining richness and abundance (i.e. the number of reads) of OTUs per sample, we also created Bray Curtis beta-diversity distance matrices between samples in *mothur*, but initial ordination plots showed no clear patterns of separation between samples based upon overall OTU communities, and thus gross beta-diversity was not investigated further. For subsequent analyses, we used both rarefied and unrarefied data sets, as appropriate (71). The former are less sensitive to contamination but more likely to have lost real, but rare OTUs. Using both approaches allowed us to confirm that our results could always be obtained both ways.

We focused our subsequent analyses on the actinobacterial OTUs within the cuticular microbiome, and in particular on species of the genus *Pseudonocardia.* First we carried out an analysis of overall OTU richness using Generalized Linear Mixed Models with negative binomial errors to partition variation between host ant species and worker age (callows vs foragers) and their interaction, using colony as a random effect. We further tested whether the attine ant species with visible actinobacteria on worker propleural plates as reported in the literature (often recorded, occasionally recorded, or absent; Figure 1) differed in overall OTU richness, and we assessed whether there was a difference in richness between those species whose ancestors originated from either South America or Central and North America (Figure 1). These analyses used Generalized Linear Mixed Models with attine species and colony ID nested within attine species as random effects. Subsequently, we carried out similar analyses, but only looking at the richness of actinobacterial OTUs. These analyses of OTU richness were carried out with both the unrarefied dataset (to examine maximum sampled richness: see (71)), and the rarefied dataset (to examine OTU richness standardized for number of reads). The richness of OTUs assigned to *Pseudonocardia* could not be examined in this way, since very few OTUs were assigned to this genus (four in the unrarefied dataset and two in the rarefied dataset). When examining the datasets for the richness of actinobacterial and *Pseudonocardia* OTUs, we chose to use all count data, even though very low OTU counts may be the product of sequencing errors or cross contamination, since there was no objective way to implement a threshold, and initial analysis with an arbitrary threshold of 10 counts yielded essentially identical results (analysis not shown).

Second, to investigate the abundance of actinobacterial and *Pseudonocardia* OTUs, we carried out a similar set of Generalized Linear Models with negative binomial errors, using the sum of the reads of all actinobacterial OTUs and the sum of the reads of *Pseudonocardia* OTUs found on the surface of each ant. We again partitioned the variance in abundance between attine species and worker age, using colony ID as a random effect, but in this case it was not possible to examine the interaction between species and worker age, as there were too many combinations where no actinobacterial or *Pseudonocardia* OTUs were recorded. We also again partitioned the data according to the presence of visible actinobacteria on the propleural plates and the geographic origin of lineages in separate analyses, using attine species and colony ID nested within attine species as random effects. Ideally, these analyses should have been carried out using the rarefied dataset to standardize maximum possible abundances across samples, but this was not possible with the analysis of *Pseudonocardia* abundance, as 70% of all combinations of factors had zero abundance, so we used the unrarefied dataset in this case, where only 36% of combinations were zero. Again, we used all count data to calculate abundances.

Third, to examine the prevalence (i.e. proportional abundance) of actinobacterial and *Pseudonocardia* OTUs, we carried out a set of Generalized Linear Models with beta-binomial errors, using the proportion of all OTU reads that were assigned to Actinobacteria, and the proportion of actinobacterial reads that were assigned to *Pseudonocardia* as dependent variables, and again partitioning the variance in prevalence between attine species, worker age and their interaction, using colony ID as a random effect. We also again partitioned the data according to the presence of visible actinobacteria on the propleural plates and the geographic origin of species in separate analyses, once again using attine species and colony ID nested within attine species as random effects. We carried out these analyses using both the rarefied and unrarefied datasets, which gave very similar results. We present only the results from the unrarefied dataset, which normalizes differences in total reads between samples as the prevalence is a ratio of the number of reads of different types of OTU within a sample, and has the advantage that all OTUs, however rare, are included, that the data is not compositional across samples, and that there are fewer samples that had zero reads in the denominator for the *Pseudonocardia* prevalence, which had to be excluded from the analysis.

As a supplementary approach, to examine the differential representation of all individual OTUs based upon different types of partitioning of unrarefied samples, we used the *DESeq2* package (72) in R to analyze transitions across: (i) samples from the basal attine lineages that evolved in South America *versus* the lineages that evolved later in Central/North America (7), and (ii) samples from species where the most common *Pseudonocardia* OTU *ActAcro1* was present in the cuticular microbiome *versus* species where it was not.

For further details of these Generalized Linear Mixed Models, *DESeq* analysis and their R-scripts, see the Supplementary Materials.

We used TEM to examine the presence and growth form of bacteria on the cuticle of worker propleural plates from five attine species, representing the main branches of the attine phylogeny (Figure 1), and to trace potential evolutionary modifications in resource provisioning of cuticular Actinobacteria: *Acromyrmex echinatior*, *Paratrachymyrmex cornetzi*, *Mycetomoellerius zeteki*, *Cyphomyrmex costatus*, and *Apterostigma dentigerum*. Workers came from the original field colonies when possible, so most colonies sampled for microscopy were now lab colonies because they had been kept for 12–36 months in climate-controlled rooms in Copenhagen. Only the TEM-sampled colonies of *Ap. dentigerum* were newly field-collected. We fixed the dissected chest plate samples (details in Supplementary material) and prepared ultrathin sections for TEM (a JEOL JEM-1010). Images were then prepared and analyzed using Adobe Illustrator (2017.1.0) and Photoshop (2017.1.1) (Supplementary material). In parallel, we sequenced the cuticular microbiomes from the propleural plates of two other workers sampled simultaneously from the same two colonies per species, to support interpretation of TEM images (Figure 2), following protocols described above and in the Supplementary material.

We next compared the proportional abundances of *Pseudonocardia* in the cuticular microbiomes with their proportional abundance in the gut microbiomes from the same Panama canal zone species available from (23) (Figure 3). This analysis focused on the single shared gut symbiont OTU *ActAcro1* identified by Sapountzis, et al (24), which turned out to capture almost all aligned sequences of both Sapountzis et al (23) and the present study. This allowed us to create a reference distance matrix of *Pseudonocardia* sequences (details in Supplementary material) and to evaluate how the *Pseudonocardia* clades identified by Cafaro et al (32) related to the sequences captured by the *ActAcro1* OTU, also using publicly available *Pseudonocardia* sequences in local BLAST analyses. Finally, we analyzed whether *ActAcro1* presence/absence in the cuticular microbiomes correlated with the representation of other bacterial OTUs in the gut microbiomes (Table S11) to complement an analysis by Sapountzis et al (23) that had used unspecified actinobacterial bloom on the cuticle to investigate whether such interactions exist.

To evaluate our findings in a broader context, we performed a literature review of all published studies on the subject prior to 2019. We collated details on the studies’ methods, morphological and DNA-based identifications of Actinobacteria, and checked additional information including evidence of antibiotic production or pathogen inhibition. We used all data that were informative and identified the number of studies reporting a *Pseudonocardia* association according to an increasingly stringent set of four likelihood criteria. These were whether *Pseudonocardia* was inferred to be present by mere observation of bacterial growth, by plated culture morphology, or by DNA sequencing; how specifically sampling was carried out (from the propleural plates or elsewhere); how widely replicated sampling was, and from which population(s) in Panama or elsewhere; and whether a broader range of attine-associated actinobacterial strains was identified or only the two specific *Pseudonocardia* species known from *Acromyrmex* spp. and *P. cornetzi* (32), i.e. *P. octospinosus* (*Ps1*) and *P. echinatior* (*Ps2*). We recorded the proportion of studies that reported *Pseudonocardia* under each of these criteria, out of the total number of relevant studies, for each of the 11 attine species investigated (see Figure 4, Figure S5 and Table S12).

## Supplementary Material

pgae391_Supplementary_Data

## Data Availability

A *fasta* file containing the quality-filtered unique sequences (see Supplementary material), aligned to the Silva reference database, is available from the NIH Sequence Read Archive (https://www.ncbi.nlm.nih.gov/sra), under BioProject number PRJNA978378. The rarefied and unrarefied OTU-read tables for each sample, upon which all other analyses are based, can be found in Table S1.

## References

[1] Boomsma JJ . 2022. Domains and major transitions of social evolution. Oxford University Press.

[2] Foster KR, Wenseleers T. 2006. A general model for the evolution of mutualisms. J Evol Biol. 19:1283–1293. 16780529 10.1111/j.1420-9101.2005.01073.x

[3] Frank S . 1998. Foundations of social evolution. Princeton: Princeton University Press.

[4] Herre EA, Knowlton N, Mueller UG, Rehner SA. 1999. The evolution of mutualisms: exploring the paths between conflict and cooperation. Trends Ecol Evol. 14:49–53. 10234251 10.1016/s0169-5347(98)01529-8

[5] Schultz TR, Brady SG. 2008. Major evolutionary transitions in ant agriculture. Proc Natl Acad Sci U S A. 105:5435–5440. 18362345 10.1073/pnas.0711024105 PMC2291119

[6] Solomon SE, et al 2019. The molecular phylogenetics of *Trachymyrmex* forel ants and their fungal cultivars provide insights into the origin and coevolutionary history of ‘higher-attine’ ant agriculture. Syst Entomol. 44:939–956.

[7] Branstetter MG, et al 2017. Dry habitats were crucibles of domestication in the evolution of agriculture in ants. Proc R Soc Lond B Biol Sci. 284:20170095. 10.1098/rspb.2017.0095 PMC5394666 28404776

[8] Mueller UG, Rehner SA, Schultz TR. 1998. The evolution of agriculture in ants. Science. 281:2034–2038. 9748164 10.1126/science.281.5385.2034

[9] Cristiano MP, Cardoso DC, Sandoval-Gómez VE, Simões-Gomes FC. 2020. *Amoimyrmex* Cristiano, Cardoso & Sandoval, *gen. nov.* (Hymenoptera: Formicidae): a new genus of leaf-cutting ants revealed by multilocus molecular phylogenetic and morphological analyses. Aust Entomol. 59:643–676.

[10] Currie CR, Mueller UG, Malloch D. 1999. The agricultural pathology of ant fungus gardens. Proc Natl Acad Sci U S A. 96:7998–8002. 10393936 10.1073/pnas.96.14.7998 PMC22176

[11] de Man TJB, et al 2016. Small genome of the fungus *Escovopsis weberi*, a specialized disease agent of ant agriculture. Proc Natl Acad Sci U S A. 113:3567–3572. 26976598 10.1073/pnas.1518501113 PMC4822581

[12] Gerardo NM, Mueller UG, Price SL, Currie CR. 2004. Exploiting a mutualism: parasite specialization on cultivars within the fungus-growing ant symbiosis. Proc R Soc Lond B Biol Sci. 271:1791–1798. 10.1098/rspb.2004.2792 PMC1691791 15315894

[13] Currie CR, Scott JA, Summerbell RC, Malloch D. 1999. Fungus-growing ants use antibiotic-producing bacteria to control garden parasites. Nature. 398:701–704.

[14] Li H, et al 2018. Convergent evolution of complex structures for ant–bacterial defensive symbiosis in fungus-farming ants. Proc Natl Acad Sci U S A. 115:10720–10725. 30282739 10.1073/pnas.1809332115 PMC6196509

[15] Currie CR, et al 2003. Ancient tripartite coevolution in the attine ant-microbe symbiosis. Science. 299:386–388. 12532015 10.1126/science.1078155

[16] Mattoso TC, Moreira DDO, Samuels RI. 2012. Symbiotic bacteria on the cuticle of the leaf-cutting ant *Acromyrmex subterraneus subterraneus* protect workers from attack by entomopathogenic fungi. Biol Lett. 8:461–464. 22130174 10.1098/rsbl.2011.0963 PMC3367728

[17] Poulsen M, et al 2010. Variation in *Pseudonocardia* antibiotic defence helps govern parasite-induced morbidity in Acromyrmex leaf-cutting ants. Environ Microbiol Rep. 2:534–540. 22896766 10.1111/j.1758-2229.2009.00098.x PMC3418327

[18] Holmes NA, et al 2016. Genome analysis of two *Pseudonocardia* phylotypes associated with *Acromyrmex* leafcutter ants reveals their biosynthetic potential. Front Microbiol. 7:2073. 28082956 10.3389/fmicb.2016.02073 PMC5183585

[19] Heine D, et al 2018. Chemical warfare between leafcutter ant symbionts and a co-evolved pathogen. Nat Commun. 9:2208. 29880868 10.1038/s41467-018-04520-1 PMC5992151

[20] Currie CR . 2001. A community of ants, fungi, and bacteria: a multilateral approach to studying symbiosis. Annu Rev Microbiol. 55:357–380. 11544360 10.1146/annurev.micro.55.1.357

[21] Little AEF, Currie CR. 2007. Symbiotic complexity: discovery of a fifth symbiont in the attine ant-microbe symbiosis. Biol Lett. 3:501–504. 17686758 10.1098/rsbl.2007.0253 PMC2396185

[22] Mueller UG, Gerardo NM, Aanen DK, Six DL, Schultz TR. 2005. The evolution of agriculture in insects. Annu Rev Ecol Evol Syst. 36:563–595.

[23] Sapountzis P, Nash DR, Schiøtt M, Boomsma JJ. 2019. The evolution of abdominal microbiomes in fungus-growing ants. Mol Ecol. 28:879–899. 30411820 10.1111/mec.14931 PMC6446810

[24] Sapountzis P, et al 2015. *Acromyrmex* leaf-cutting ants have simple gut microbiota with nitrogen-fixing potential. Appl Environ Microbiol. 81:5527–5537. 26048932 10.1128/AEM.00961-15 PMC4510174

[25] Sapountzis P, Zhukova M, Shik JZ, Schiott M, Boomsma JJ. 2018. Reconstructing the functions of endosymbiotic Mollicutes in fungus-growing ants. eLife. 7:e39209. 30454555 10.7554/eLife.39209 PMC6245734

[26] Zhukova M, Sapountzis P, Schiøtt M, Boomsma J. 2022. Phylogenomic analysis and metabolic role reconstruction of mutualistic Rhizobiales hindgut symbionts of Acromyrmex leaf-cutting ants. FEMS Microbiol Ecol. 98:fiac084 . 35906195 10.1093/femsec/fiac084

[27] Hanisch PE, Sosa-Calvo J, Schultz TR. 2022. The last piece of the puzzle? Phylogenetic position and natural history of the monotypic fungus-farming ant genus *Paramycetophylax* (Formicidae: Attini). Insect Syst Divers. 6(1):1–17.

[28] Villesen P, Mueller UG, Schultz TR, Adams RMM, Bouck AC. 2004. Evolution of ant-cultivar specialization and cultivar switching in *Apterostigma* fungus-growing ants. Evolution. 58:2252–2265. 15562688 10.1111/j.0014-3820.2004.tb01601.x

[29] Dentinger B, Lodge D, Munkacsi A, Desjardin D, McLaughlin D. 2009. Phylogenetic placement of an unusual coral mushroom challenges the classic hypothesis of strict coevolution in the *Apterostigma pilosum* group ant-fungus mutualism. Evolution. 63:2172–2178. 19453731 10.1111/j.1558-5646.2009.00697.x

[30] Mueller UG, Dash D, Rabeling C, Rodrigues A. 2008. Coevolution between attine ants and actinomycete bacteria: a reevalutaion. Evolution. 62:2894–2912. 18752608 10.1111/j.1558-5646.2008.00501.x

[31] Sen R, et al 2009. Generalized antifungal activity and 454-screening of *Pseudonocardia* and *Amycolatopsis* bacteria in nests of fungus-growing ants. Proc Natl Acad Sci U S A. 106:17805–17810. 19805175 10.1073/pnas.0904827106 PMC2764928

[32] Cafaro MJ, et al 2011. Specificity in the symbiotic association between fungus-growing ants and protective *Pseudonocardia* bacteria. Proc R Soc Lond B Biol Sci. 278:1814–1822. 10.1098/rspb.2010.2118 PMC3097832 21106596

[33] Poulsen M, Cafaro M, Boomsma JJ, Currie CR. 2005. Specificity of the mutualistic association between actinomycete bacteria and two sympatric species of *Acromyrmex* leaf-cutting ants. Mol Ecol. 14:3597–3604. 16156826 10.1111/j.1365-294X.2005.02695.x

[34] Andersen SB, Hansen LH, Sapountzis P, Sorensen SJ, Boomsma JJ. 2013. Specificity and stability of the *Acromyrmex-Pseudonocardia* symbiosis. Mol Ecol. 22:4307–4321. 23899369 10.1111/mec.12380 PMC4228762

[35] Barrera CA, Sosa-Calvo J, Schultz TR, Rabeling C, Bacci M Jr. 2022. Phylogenomic reconstruction reveals new insights into the evolution and biogeography of Atta leaf-cutting ants (Hymenoptera: Formicidae). Syst Entomol. 47:13–35.

[36] Morrone JJ . 2006. Biogeographic areas and transition zones of Latin America and the Caribbean islands based on panbiogeographic and cladistic analyses of the entomofauna. Annu Rev Entomol. 51:467–494. 16332220 10.1146/annurev.ento.50.071803.130447

[37] Barke J, et al 2010. A mixed community of actinomycetes produce multiple antibiotics for the fungus farming ant *Acromyrmex octospinosus*. BMC Biol. 8:109. 20796277 10.1186/1741-7007-8-109 PMC2942817

[38] Seipke RF, et al 2011. A single *Streptomyces* symbiont makes multiple antifungals to support the fungus farming ant *Acromyrmex octospinosus*. PLoS One. 6:e22028. 21857911 10.1371/journal.pone.0022028 PMC3153929

[39] Marsh SE, et al 2013. Association between *Pseudonocardia* symbionts and *Atta* leaf-cutting ants suggested by improved isolation methods. Int Microbiol. 16:17–25. 24151778 10.2436/20.1501.01.176

[40] Kost C, et al 2007. Non-specific association between filamentous bacteria and fungus-growing ants. Naturwissenschaften. 94:821–828. 17541536 10.1007/s00114-007-0262-y

[41] Cafaro MJ, Currie CR. 2005. Phylogenetic analysis of mutualistic filamentous bacteria associated with fungus-growing ants. Can J Microbiol. 51:441–446. 16121221 10.1139/w05-023

[42] Meirelles LA, et al 2014. Broad *Escovopsis*-inhibition activity of *Pseudonocardia* associated with *Trachymyrmex* ants. Environ Microbiol Rep. 6:339–345. 24992532 10.1111/1758-2229.12132

[43] Poulsen M, Erhardt DP, Molinaro DJ, Lin T-L, Currie CR. 2007. Antagonistic bacterial bnteractions help shape host-symbiont dynamics within the fungus-growing ant-microbe mutualism. PLoS One. 2:e960. 17896000 10.1371/journal.pone.0000960 PMC1978530

[44] Currie CR, Poulsen M, Mendenhall J, Boomsma JJ, Billen J. 2006. Coevolved crypts and exocrine glands support mutualistic bacteria in fungus-growing ants. Science. 311:81–83. 16400148 10.1126/science.1119744

[45] Sosa-Calvo J, et al 2013. *Cyatta abscondita*: taxonomy, evolution, and natural history of a new fungus-farming ant genus from Brazil. PLoS One. 8:e80498. 24260403 10.1371/journal.pone.0080498 PMC3829880

[46] Fernandez-Marin H, Zimmerman JK, Nash DR, Boomsma JJ, Wcislo WT. 2009. Reduced biological control and enhanced chemical pest management in the evolution of fungus farming in ants. Proc R Soc Lond B Biol Sci. 276:2263–2269. 10.1098/rspb.2009.0184 PMC2677613 19324734

[47] Fernandez-Marin H, Zimmerman JK, Rehner SA, Wcislo WT. 2006. Active use of the metapleural glands by ants in controlling fungal infection. Proc R Soc Lond B Biol Sci. 273:1689–1695. 10.1098/rspb.2006.3492 PMC1634922 16769642

[48] Fernandez-Marin H, et al 2015. Functional role of phenylacetic acid from metapleural gland secretions in controlling fungal pathogens in evolutionarily derived leaf-cutting ants. Proc R Soc Lond B Biol Sci. 282:20150212. 10.1098/rspb.2015.0212 PMC4424645 25925100

[49] Haeder S, Wirth R, Herz H, Spiteller D. 2009. Candicidin-producing *Streptomyces* support leaf-cutting ants to protect their fungus garden against the pathogenic fungus *Escovopsis*. Proc Natl Acad Sci U S A. 106:4742–4746. 19270078 10.1073/pnas.0812082106 PMC2660719

[50] Kim J, et al 2023. *Amycolatopsis* from desert specialist fungus-growing ants suppresses contaminant fungi using the antibiotic ECO-0501. Appl Environ Microbiol. 89:e0183822. 36700628 10.1128/aem.01838-22 PMC9972958

[51] van Keulen G, Hopwood DA, Dijkhuizen L, Sawers RG. 2005. Gas vesicles in actinomycetes: old buoys in novel habitats? Trends Microbiol. 13:350–354. 15993071 10.1016/j.tim.2005.06.006

[52] Bacon CD, et al 2015. Biological evidence supports an early and complex emergence of the Isthmus of Panama. Proc Natl Acad Sci U S A. 112:6110–6115. 25918375 10.1073/pnas.1423853112 PMC4434730

[53] Munkacsi AB, et al 2004. Convergent coevolution in the domestication of coral mushrooms by fungus–growing ants. Proc R Soc Lond B Biol Sci. 271:1777–1782. 10.1098/rspb.2004.2759 PMC1691797 15315892

[54] Schultz TR, et al 2015. The most relictual fungus-farming ant species cultivates the most recently evolved and highly domesticated fungal symbiont species. Am Nat. 185:693–703. 25905511 10.1086/680501

[55] Seipke RF, Barke J, Heavens D, Yu DW, Hutchings MI. 2013. Analysis of the bacterial communities associated with two ant–plant symbioses. Microbiologyopen. 2:276–283. 23417898 10.1002/mbo3.73 PMC3633351

[56] Scheuring I, Yu DW. 2012. How to assemble a beneficial microbiome in three easy steps. Ecol Lett. 15:1300–1307. 22913725 10.1111/j.1461-0248.2012.01853.x PMC3507015

[57] Currie CR, Bot ANM, Boomsma JJ. 2003. Experimental evidence of a tripartite mutualism: bacteria protect ant fungus gardens from specialized parasites. Oikos. 101:91–102.

[58] Worsley SF, et al 2021. Competition-based screening helps to secure the evolutionary stability of a defensive microbiome. BMC Biol. 19:205. 34526023 10.1186/s12915-021-01142-w PMC8444595

[59] Andersen S, Yek S, Nash D, Boomsma J. 2015. Interaction specificity between leaf-cutting ants and vertically transmitted *Pseudonocardia* bacteria. BMC Evol Biol. 15:27. 25886448 10.1186/s12862-015-0308-2 PMC4346108

[60] Barka EA, et al 2016. Taxonomy, physiology, and natural products of Actinobacteria. Microbiol Mol Biol Rev. 80:1–43. 26609051 10.1128/MMBR.00019-15 PMC4711186

[61] Martin M . 1987. Invertebrate-microbial interactions: ingested fungal enzymes in arthropod biology. In: Eisner T, Meinwald J, editors. Explorations in chemical ecology. Ithaca (NY): Cornell University Press.

[62] Schiøtt M, Boomsma J. 2021. Proteomics reveals synergy between biomass degrading enzymes and inorganic Fenton chemistry in leaf-cutting ant colonies. Elife. 10:e61816. 33433325 10.7554/eLife.61816 PMC7877906

[63] Fernández-Marín H, et al 2013. Dynamic disease management in *Trachymyrmex* fungus-growing ants (Attini: Formicidae). Am Nat. 181:571–582. 23535621 10.1086/669664

[64] Gotting K, et al 2022. Genomic diversification of the specialized parasite of the fungus-growing ant symbiosis. Proc Natl Acad Sci U S A. 119:e2213096119. 36508678 10.1073/pnas.2213096119 PMC9907069

[65] Nygaard S, et al 2016. Reciprocal genomic evolution in the ant-fungus agricultural symbiosis. Nat Commun. 7:12233. 27436133 10.1038/ncomms12233 PMC4961791

[66] Schiøtt M, Rogowska-Wrzesinska A, Roepstorff P, Boomsma JJ. 2010. Leaf-cutting ant fungi produce cell wall degrading pectinase complexes reminiscent of phytopathogenic fungi. BMC Biol. 8:156. 21194476 10.1186/1741-7007-8-156 PMC3022778

[67] Beasley DE, Koltz AM, Lambert JE, Fierer N, Dunn RR. 2015. The evolution of stomach acidity and its relevance to the human microbiome. PLoS One. 10:e0134116. 26222383 10.1371/journal.pone.0134116 PMC4519257

[68] Caporaso JG, et al 2011. Global patterns of 16S rRNA diversity at a depth of millions of sequences per sample. Proc Natl Acad Sci U S A. 108:4516–4522. 20534432 10.1073/pnas.1000080107 PMC3063599

[69] Kozich JJ, Westcott SL, Baxter NT, Highlander SK, Schloss PD. 2013. Development of a dual-index sequencing strategy and curation pipeline for analyzing amplicon sequence data on the MiSeq illumina sequencing platform. Appl Environ Microbiol. 79:5112–5120. 23793624 10.1128/AEM.01043-13 PMC3753973

[70] Quast C, et al 2013. The SILVA ribosomal RNA gene database project: improved data processing and web-based tools. Nucleic Acids Res. 41:D590–D596. 23193283 10.1093/nar/gks1219 PMC3531112

[71] McMurdie PJ, Holmes S. 2014. Waste not, want not: why rarefying microbiome data is inadmissible. PLoS Comput Biol. 10:e1003531. 24699258 10.1371/journal.pcbi.1003531 PMC3974642

[72] Love MI, Huber W, Anders S. 2014. Moderated estimation of fold change and dispersion for RNA-Seq data with DESeq2. Genome Biol. 15:550. 25516281 10.1186/s13059-014-0550-8 PMC4302049

