# Supplementary material for: From the inside out: Were the cuticular Pseudonocardia bacteria of fungus-farming ants originally domesticated as gut symbionts?
Source: PNAS Nexus. 2024 Oct 15;3(10):pgae391. doi: 10.1093/pnasnexus/pgae391 (PMC11474983; doi:10.1093/pnasnexus/pgae391)
Supplement: pgae391_Supplementary_Data [file pgae391_supplementary_data.pdf]

Supplementary Material

From the inside out: were the cuticular *Pseudonocardia* bacteria of fungus-farming ants originally domesticated as gut symbionts?

Tabitha M. Innocent, Panagiotis Sapountzis*, Mariya Zhukova, Michael Poulsen, Morten Schiøtt, David R. Nash* and Jacobus J. Boomsma*

***Correspondence** panagiotis.sapountzis@inrae.fr, drnash@bio.ku.dk, jjboomsma@bio.ku.dk

1 Supplementary Methods and Results

1.1 Field collections and sampling

We sampled ants from 11 attine species in the Gamboa area of Soberania National Park, which represent all nine of the currently described genera that can be found in the Panama Canal Zone (see Figure 1 for attine phylogeny). We collected 4 - 11 colonies per species, with the exception of *Myrmicocrypta ednaella* for which we found only a single colony. After returning to the field station, we retained 62 colonies that had sufficient biomass to allow effective sampling. We then removed individual ants from their colonies, placing 4-6 young callow workers (lighter pigmentation of cuticle, always located inside the fungus garden) and 4-6 older foraging workers (darker cuticle, outside the fungus garden) individually into 2 ml collection tubes filled with 99% ethanol using flame-sterilized forceps. For those species previously reported to maintain visible actinobacterial blooms consistently and abundantly on the propleural chest plates, we made sure the sampled workers had the typical white bloom on the chest plates or, as occasionally reported (1, 2), under the forelegs in some of the phylogenetically most basal attine species.

1.2 Dissection, DNA extraction, sequencing

We normally used four workers per colony – two callows and two foragers – for each species (fewer in 13 cases where only a single worker for one or both age classes was present in the colony), giving a total of 226 individuals. We dissected each ant to remove the propleural (chest) plates under sterile bench conditions using sterilized forceps and a stereomicroscope, and placed these individually in sterile 2 ml vials, which were stored at -20°C until DNA extraction.

We extracted DNA from each individual ant sample using the Qiagen Blood & Tissue DNA kit following the manufacturers' specified protocol, with the additional step of vortexing each sample for 30 seconds with 0.1 mm glass beads at the first stage of DNA extraction. Each sample was re-eluted in 100 µl AE elution buffer, as were three negative extractions as controls. After extraction, we ran 16S PCR reactions using universal primers (341F/806R) (2, 3) to identify the optimal conditions/templates for library preparation, and to confirm that there was no detectable DNA amplification in the negative DNA extractions. We sent all extracted DNA samples and blank controls to the Microbial Systems Laboratory at the University of Michigan for library preparation and Illumina MiSeq sequencing, where two water samples were included in the MiSeq sequencing run as further controls (protocol described in (4), see also (5)).

1.3 Data analysis

We analyzed the raw sequence data in *mothur* (v 1.36.1; mothur.org, page first accessed 27th July 2015; last accessed October 2017) using the standard operating procedure developed for Illumina MiSeq data with modifications as described previously (5). Sequences were assembled using the *make.contigs* command. We then trimmed, filtered and identified unique sequences before aligning them using the SILVA 111 non-redundant database (6). Sequences or parts of the sequences that did not cover positions 13862-23444 of the V4 region of the SILVA alignment or had a homopolymer length of 8 or above were removed, as were chimeric sequences. Remaining sequences were classified (Bayesian classifier implemented in *mothur*) using the same database (SILVA), based on an 80% confidence threshold. During the filtering steps we adapted the *remove.lineage* command to remove extraneous sequences from mitochondria, chloroplasts, archaea and eukaryotes. We also removed

Wolbachia sequences before rarefaction and further analysis, because these endosymbionts occur only in *Acromyrmex* and *Apterostigma*, occasionally reaching very high prevalences without being parasites in *Acromyrmex*, and would thus have biased our general comparisons across all attine ants (see 3, 7). OTUs were identified using a 97% sequence similarity criterion.

We rarefied the data to standardize for the number of reads per sample, first by generating pseudo-datasets with between 1 and 82,000 reads, and then by simulating 1000 iterations per replicate and visualizing the rarefaction curves in Microsoft Excel (2011 version 14.6.8 for Mac). Rarefied data were examined for under-sampling, which led us to remove 105 of the 299 samples, producing a OTU dataset rarefied to 2000 reads. This reduced the final number of OTUs from 508 to 247 (Table S1), and we used these for subsequent analyses unless otherwise specified. Alongside the efforts made to avoid any contamination throughout the extraction and sequencing process, we included two blank (water) samples in the same MiSeq run. These produced very few reads, but any sequences from these blanks that were found in the actual samples were also removed when the ratio of water:ant relative abundance exceeded 0.2 (7, 8).

1.4 Pseudonocardia species associated with **Acromyrmex** *leaf-cutting ants in Panama*

Attine-associated *Pseudonocardia* were first described in detail for the leaf-cutting ant genus *Acromyrmex*, with two species, *Ps1* and *Ps2*, occurring at approximately equal frequency in the Panamanian field population (the same site as the present study where *A. echinatior* and *A. octospinosus* coexist), with large workers from any given colony carrying one of these two strains (3, 9). The sequenced genomes of five *Pseudonocardia* isolates each of *Ps1* and *Ps2* from different colonies at the same Gamboa site have recently been obtained (10). Subsequent genome comparison and the identification of secondary metabolite (SMB) clusters (regions encoding the production of antibiotics and other biologically active compounds) showed that these two strains were true species, so they were named *P. octospinosus* (*Ps1*) and *P. echinatior* (*Ps2*) (10). While both strains share a number of SMB clusters, they also have unique species-specific sets (shared by some or all of the five representative isolates of the same *Pseudonocardia* species sequenced but absent in all isolates of the other species). It is likely that these secondary antibacterial metabolites are important both for defense against pathogens and in competition among bacterial strains for niche space on the ant cuticle (11, 12). The two *Pseudonocardia* species also produce a single antifungal metabolite each, nystatin-P1 (*P. octospinosus*) and a nystatin-like polyene (*P. echinatior*), which are likely to be important in the biocontrol of the fungal pathogen *Escovopsis* that threatens the mutualistic fungus gardens of the ants (10).

1.5 Statistical analysis

Overall OTU richness per sample was calculated from the rarefied dataset (Table S1), as were the richnesses of Actinobacteria and of Pseudonocardiaceae OTUs. We originally created overall Bray-Curtis beta-diversity distance matrices in mothur, but initial ordination (NMDS) plots showed no clear patterns of separation between samples based upon ant species. This was generally confirmed by PERMANOVA, which showed only significant separation of total cuticular microbiomes between *A. echinatior* and *S. amabilis* and between *A. octospinosus* and *M. smithii*. Gross overall compositional differences in cuticular bacterial communities were therefore not investigated further, but we instead focused on the relative composition of *Pseudonocardia* and actinobacteria among the total cuticular microbiome.

Since the richness, abundance and prevalence (see precise definitions below) of both the actinobacteria and Pseudonocardia were very variable between samples, with many samples with zero values (i.e. the data were “sparse”), we analyzed their distribution between samples using Generalized Linear Mixed Models (GLMMs) implemented in the R package *glmmTMB*, which performs well with sparse and zero-inflated data (13), using both either the rarefied or unrarefied data or both, as appropriate.

Richness was defined as the number of distinct OTUs (overall, or belonging to actinobacteria) detected in each sample. How this varied between samples was analyzed using a GLMM with negative binomial error structure. For the initial analysis, ant host species (n = 11), worker caste (callow or forager) and their interaction were treated as fixed main effects, with ant colony identity treated as a random effect. The significance of the main effects was assessed using Wald Chi-squared tests as implemented in *glmmTMB*, with post-hoc tests between different ant species based on the Šídák method, as implemented in the *cld* command of the *emmeans* package (14). We also examined the variation in richness between samples partitioned in two additional ways based upon species characteristics;

species that were recorded as having visible (actinobacterial) blooms present on the chest plates or between the front legs (coded as three categories: *Present*: Blooms usually present, *Sparse*: Blooms occasionally present, and *Absent*: Blooms absent; see Table S1), and between those ant species that had their most likely geographic origin in Central or North America (*Nearctic*) versus those with a likely origin in South America (*Neotropical*) (15). In both these cases, the partition was examined as a main effect, with ant species, and ant colony ID nested within ant species as random effects. Wald Chi-squared tests were used to examine main effects. The use of the rarefied or unrarefied data sets had different implications on measures of richness, since the unrarefied dataset was more likely to contain OTUs represented by one or a few counts, particularly for samples with a high overall count. Rarefying to 2000 reads reduced the potential effects of low read counts that may have represented artefacts, but meant that some low abundance OTUs were excluded that may have been relevant. Both sets of analysis are therefore potentially useful. *Pseudonocardia* richness was not examined because of the low number of *Pseudonocardia* OTUs identified (Two in the rarefied dataset, four in the unrarefied daatset) Results are presented in table S2.

Abundance was defined as the sum of the reads of relevant OTUs in each sample, i.e. the sum of the reads of all actinobacterial OTUs or *Pseudonocardia* OTUs. This was analyzed using GLMMs in the same way as richness, also using a negative binomial error structure, and with the same sets of independent variables. The use of the rarefied or unrarefied data sets had different implications on measures of abundance, with the rarefied dataset giving a measure of relative abundance based on an overall abundance of 2000. The unrarefied abundance also incorporated between sample differences in "sampling" (including potential. Artefacts), but also included all relatively rare actinobacterial OTUs. Generally speaking, the results of tests of abundance based on the rarefied dataset are expected to be referred to those on the unrarefied dataset. Results are presented in table S4.

Prevalence was defined as proportion of all OTU reads that were actinobacterial (i.e. actinobacterial abundance divided by the sum of all reads) or the proportion of all actinobacterial reads that were Pseudonocardia (i.e. Pseudonocardia abundance divided by actinobacterial abundance) in each sample. This was analyzed using GLMMs in a similar way as richness and abundance, with the same sets of independent variables, but in this case a beta-binomial error structure was used. The use of the rarefied or unrarefied data sets had slightly different implications on measures of prevalence, although since they represent proportional data, the results should be comparable. As for richness, the main difference between the datasets will be in the inclusion (and abundance) or relatively rare, but potentially important, OTUs. Both sets of analysis are therefore potentially useful, but that based on the unrarefied dataset has several advantages; while the data are compositional within samples, the denominators vary, which removes many of the problems associated with analyzing compositional data. The number of denominators in the Pseudonocardia prevalence dataset is also smaller in the unrarefied dataset, which means the number of samples that have to be excluded because of this is lower, giving greater statistical power. Results are presented in table S5.

To examine the differential representation of all individual OTUs based upon different types of partitioning of unrarefied samples, we used the *DESeq2* package in R (16) to analyze transitions across: (i) samples from the basal attine lineages that evolved in South America *versus* the lineages that evolved later in Central/North America (15), and (ii) samples from species where the *Pseudonocardia* OTU *ActAcro1* was present in the cuticular microbiome *versus* species where it was not (Table S11).

We manually inspected the unrarefied data across all samples to see whether any reads from well-known actinobacterial genera such as *Streptomyces* and *Amycolatopsis* were present in such low abundances that they were not retained in the rarefied data set. This revealed a total of 26 Actinobacterial OTUs that were present sparsely and at low abundance in the dataset, including two additional Pseudonocardiaceae (Table S1B), but no *Streptomyces* or *Amycolatopsis*.

1.6 Microscopy

To examine the presence and growth forms of bacteria on the cuticle of the propleural chest plates, we selected five ant species representing the different main branches of the attine phylogeny (see Figure 1): *Acromyrmex echinatior*, *Paratrachymyrmex cornetzi*, *Mycetomoellerius zeteki*, *Cyphomyrmex costatus* and *Apterostigma dentigerum*. We sampled two lab colonies for each ant species, taking several mature callow workers from the fungus garden with visible actinobacterial blooms on their chest plates. As far as possible, we used colonies from the original collection (Table S1), substituting with later-collected colonies only when original colonies had not survived in the lab during the 36 months between field collection and lab sampling. These time lapses meant that we obtained lab-samples for

the *Acromyrmex*, *Mycetomoellerius* and *Cyphomyrmex* colonies (all from the original field colonies) and for *Paratrachymyrmex* (where we sampled colonies collected 24 months later at the same field site that were kept in the lab for 12 rather than 36 months), but we used new field samples for *Apterostigma* colonies from May 2017 to obtain sections from the complete mesothorax focusing particularly on the middle groove. This distinction is relevant because lab sampled ants have been shown to often carry secondarily acquired actinobacteria in their cuticular microbiota that are not found in the field (7).

We dissected propleural chest plates of ants from all five species, plus the mesothorax for four additional *A. dentigerum* workers, in 0.01 M phosphate buffer (pH 7.4) and then fixed material in a solution of 2.5 % glutaraldehyde (Sigma) in 0.1 M sodium cacodylate buffer (pH 7.4) for 2.5 hours. We subsequently washed tissues with the same buffer and immersed them in a post-fixation solution of 1 % osmium tetroxide (OsO_4, in sodium cacodylate buffer) for 1 hour before placing samples in a 1 % aqueous solution of uranyl acetate kept at 4°C overnight. Next, we dehydrated samples in an ethanol series and acetone, and embedded them in Spurr low-viscosity resin (Ted Pella Inc.). We stained ultrathin sections with uranyl acetate and Reynolds lead citrate and examined these with a transmission electron microscope (JEM 1010, JEOL). Images were analyzed and accompanying illustrations created using Adobe Illustrator (2017.1.0) and Photoshop (2017.1.1) (Figures 2, S1, S2, S3).

Two additional individuals per colony were removed at the same time as the ones used for microscopy, and had their propleural chest plates dissected and cuticular microbiomes sequenced, using the same protocol as described above, to give information on the identity of bacteria present from directly comparable samples to substantiate the interpretation based upon bacterial structures inferred from microscopy (see Table S6 for relative abundances of actinobacterial OTUs; Table S7 for comparison between these predominantly lab samples and earlier field collections).

1.7 Comparing the microbiomes of attine ant guts and cuticles

We compared the proportional abundance of *Pseudonocardia* in the cuticular microbiomes with the proportional abundance in the gut microbiomes across the attine phylogeny. To do this, we extracted data on the sequence identity and relative abundances of *Pseudonocardia* within cuticular microbiomes (this study) and the gut microbiomes (7) for the 11 attine species included in both studies (Table S8), focusing on the single shared *Pseudonocardia* OTU designated as *ActAcro1* by Sapountzis et al. (7, 17). Once we established that the *ActAcro1* OTU was identical in both studies (by comparing both representative OTU sequences generated with the *get.oturep* command in mothur, and the unique sequences associated with them), the comparison of mean relative abundances of reads for each host ant species within this OTU produced a remarkable pattern of mutual exclusiveness (Figure 3). To obtain the sequences we needed for comparisons within the *ActAcro1* OTU, we used the *fasta* file that was created for each dataset ('gut' or 'cuticle') with 'mothur' after quality filtering and classification, which contained the unique sequences that were used for clustering the OTUs, to create a BLAST database using the *makeblastdb* command in *blastn* (18). Using the representative 16S sequence of the *ActAcro1* OTU that had been identified in a previous study (17), we extracted all sequences from each dataset that showed ≥97% similarity with *ActAcro1* along with information about their relative abundances and the ant samples from which they originated. We then aligned these sequences using MUSCLE implemented in Geneious R7.1 to construct a distance matrix, which we imported in Excel 2013 to generate a heatmap (Table S10).

To establish how the *Pseudonocardia* clades previously identified by Cafaro et al. (19) relate to the *ActAcro1* OTU identified in our dataset for cuticular and gut microbiomes, we downloaded all *Pseudonocardia* sequences publicly available on NCBI from Cafaro et al. (19), merged them into a single *fasta* file and created a BLAST database using the *makeblastdb* command. We then performed local BLASTs to compare the 97% identity *ActAcro1* OTU with the Cafaro sequences (Table S9).

To examine whether the cuticular bacterial community had any association with the gut bacterial community, we used the DESeq2 program in R (Love et al., 2014) to perform a differential representation analysis. Here, gut microbiome samples (acquired from (7) were split based on whether the OTU *ActAcro1* was present or absent in the cuticular microbiome of the same ant host species in the present study, to test whether the gut microbial representation of this OTUs was positively or negatively correlated in comparisons across host ant species (Table S11). This analysis is similar to an analysis carried out by Sapountzis et al. (7), where methods are detailed), but we now grouped samples specifically based upon the documented presence/absence of *ActAcro1*, rather than on the mere presence/absence of a visible but unspecified actinobacterial 'bloom' on the propleural chest plates of workers based on the literature.

1.8 Literature review of previous studies on attine-ant-associated **Pseudonocardia**

The role of *Pseudonocardia* as a symbiont on the cuticle has been a focus of attine research over the last two decades, and also a source of controversy (1-3, 9, 20-26). We therefore carried out an extensive literature review of all published studies looking for attine-associated *Pseudonocardia* to reconstruct the extent to which our empirical results aligned with findings of previous work. As the Sapountzis et al. (2015, 2018*a*) studies were the first to look at *Pseudonocardia* in the attine gut microbiomes, our survey only looked for published data on attine cuticular microbiomes. Our analysis was completed in November 2017, so did not include a recent study by Li et al. (27), except that we comment on that study in a few places.

We collected a full sample of publications based on Google scholar/Web of Science keyword searches, supplemented with tracking older references to make sure all relevant studies were included in our comparisons. For each published study we scrutinized data tables, method sections and supplementary files for information on: 1. the attine genera and species studied; the methods used for collection and sampling; the scope of sampling (numbers of colonies and individuals collected and analyzed, and which tissue(s) were sampled; 2. any morphological description of bacterial 'bloom' and cuticular locations; 3. any information on the presence/absence of *Pseudonocardia* and any other actinobacterial species (e.g. *Streptomyces* or *Amycolatopsis*); 4. any additionally relevant details, such as evidence of pathogen inhibition, or the production/activity of antibacterial compounds (Table S12). We used all these informative data pertaining specifically to attine ant workers for direct comparison with the results of our present study (Table S12). We organized records by attine genera and species, and looked at the number of studies reporting an association with *Pseudonocardia*, given an increasingly stringent set of criteria that we used when tabulating the results: whether *Pseudonocardia* was identified by observation of bacterial growth, culture morphology, or DNA sequencing; how specifically sampling was carried out; how widely replicated sampling was and from which population(s); and whether a broader range of attine-associated strains or just the two specifically described species known to be associated with *Acromyrmex* leaf-cutting ants (*P. octospinosus* and *P. echinatior*) were identified (see Figure 4, Figure S5 and Table S12). We calculated the proportion of studies – for each of the 11 attine species considered in our present study – that reported *Pseudonocardia* under each of these criteria, out of the total number of 25 relevant previous studies (see Figure 4; also Figure S5 and Table S12).

2 Supplementary discussion

2.1 Aerobic/anaerobic potential of Actinobacteria

The occurrence of closely related *Pseudonocardia* species on the cuticular propleural plates and in the guts of attine ants suggests that genes allowing growth with and without oxygen can be lost or gained via horizontal transmission, and maintained depending on selection pressure justifying or precluding the cost of expression of these genes. We hypothesize that substantial variation for aerobic/anaerobic phenotypes within the same 97% identity OTU is likely to be present, but obtaining data on this question was beyond the scope of this study. However, there are a number of previous studies that make us confident that this lack of data will not compromise our conclusions. The Actinobacteria in general are mostly aerobic, but exceptions do occur (28) and some strains have been documented to be facultative anaerobes (29) or to be able to grow under microaerophilic conditions (30). This pattern has also been found within the genus *Pseudonocardia* (31), so it seems reasonable to assume such variation also exists within the *ActAcro1* OTU (17) that captures essentially all Panamanian representatives of the attine-ant associated *Pseudonocardia* clades I-VI (19; Figure S5, Table S9). Further research sequencing genomes of *Pseudonocardia* strains from attine ant guts will be needed to validate the mechanisms by which these symbiotic bacteria are able to grow under oxygen-deficient conditions in the ant guts. The distribution patterns of attine-associated Actinobacteria uncovered to date (Figures 3, S5; see also (17, 19)) justify the hypothesis that the basal *Pseudonocardia* branches will predominantly be obligate or facultative anaerobes when they remain restricted to life in the gut microbiomes of phylogenetically basal attine ants.

3 Supplementary Figures and Tables

3.1 Supplementary Figures

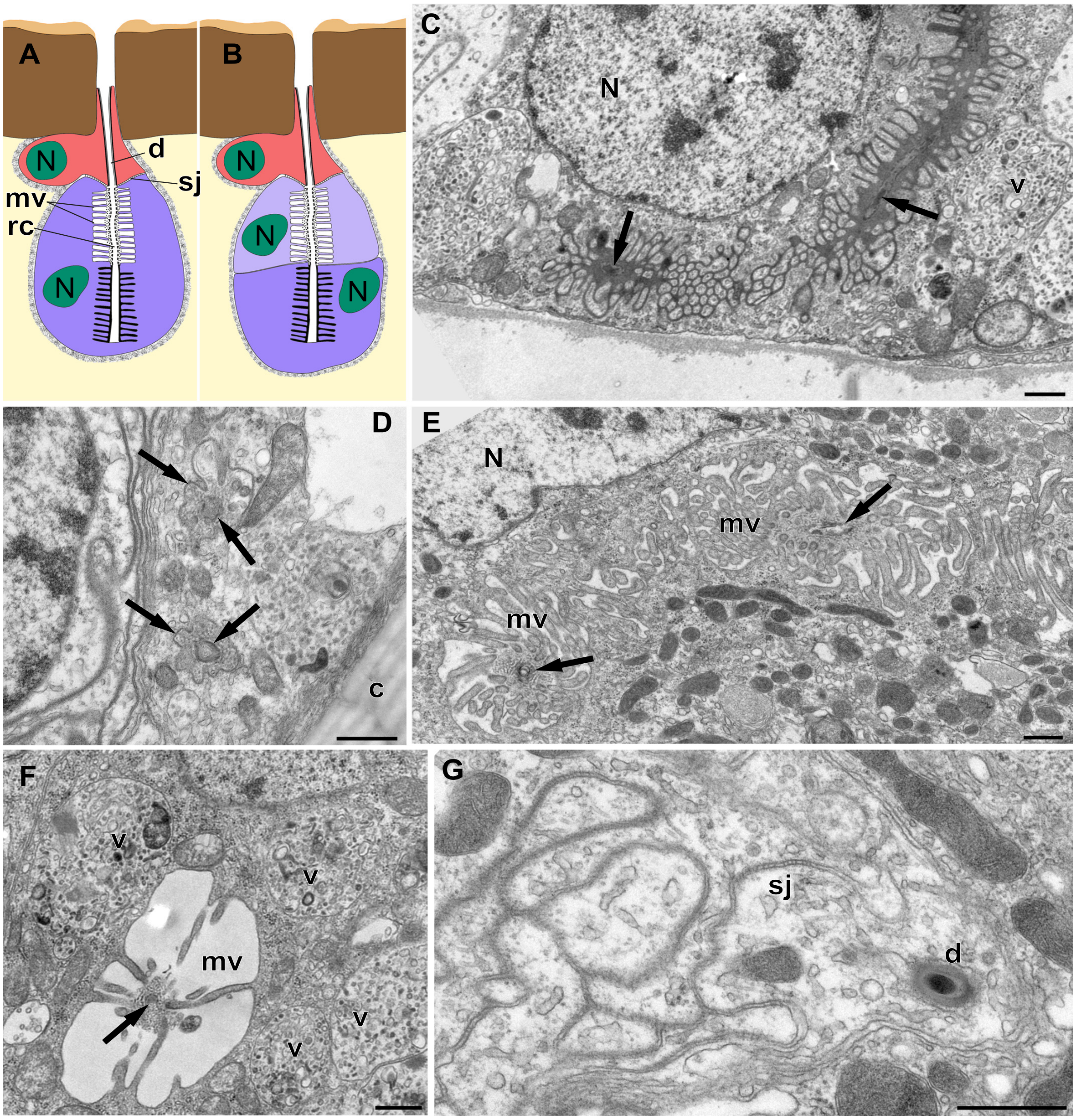

Figure S1. Further illustration of the structural complexity of the exocrine glands (class 3 in the scheme of (34)) lying below the cuticle of *A. echinatior* workers where these glands became tricellular. It appears that the glands in the rather recently evolved leaf-cutting ants with their many derived social and symbiotic traits are slightly more elaborated than the propleural chest plate glands in *P. cornetzi* (Figure 2). Comparing these two types of gland appears to leave no doubt that they are homologous because they share the same bauplan. The only difference is that the *Acromyrmex* glands have two rather than one type of canal in the secretory cells, affecting the interface with the receiving canal, which either consists of a circular arrangement of microvilli (*P. cornetzi*; Figure 2) or an extra row of parallel invaginations of the plasma membrane that are morphologically different from the microvilli. Our data do not provide information on how the two types of receiving canal connect to each other (in one or two different cells), but we assumed that in a structural sense the parallel invaginations cannot have excretory capacity comparable to microvilli and should thus be part of the gland while microvilli around the receiving canal participate in excretion. The most parsimonious explanation is that the *P. cornetzi* gland represents the ancestral state of the Neoattines because it is identical to the *M. zeteki* and *C. costatus* glands (Figure S2) and that the *Acromyrmex* gland is evolutionarily derived. A remaining unanswered question is whether the secretory cell split into two cells (blue and violet) after (**A**) or before (**B**) the microvilli (white) changed into serial invaginations (black) in the lower (part of the) cell. Panels **C-G** illustrate the key morphological features using TEM: (**C**) a receiving canal (arrow) surrounded by the plasma membrane with abundant invaginations; (**D-F**) receiving canals (arrows) surrounded by a microvillous border developed to different extents – arrowheads in (**D**) denote sections through the receiving canal with only weakly pronounced surrounding microvilli; (**G**) a duct cell (red in panel A and B). Abbreviations denote: the nucleus (**N**), microvilli (**mv**), receiving canal (**rc**), duct (**d**); cuticle (**c**), septate junction (**sj**), vesicles with heterogeneous inclusions, which might be related to secretory activity of the cells (**v**). Scale bars are 0.5 µm. The cuticle is drawn in dark brown as in Figure 2.

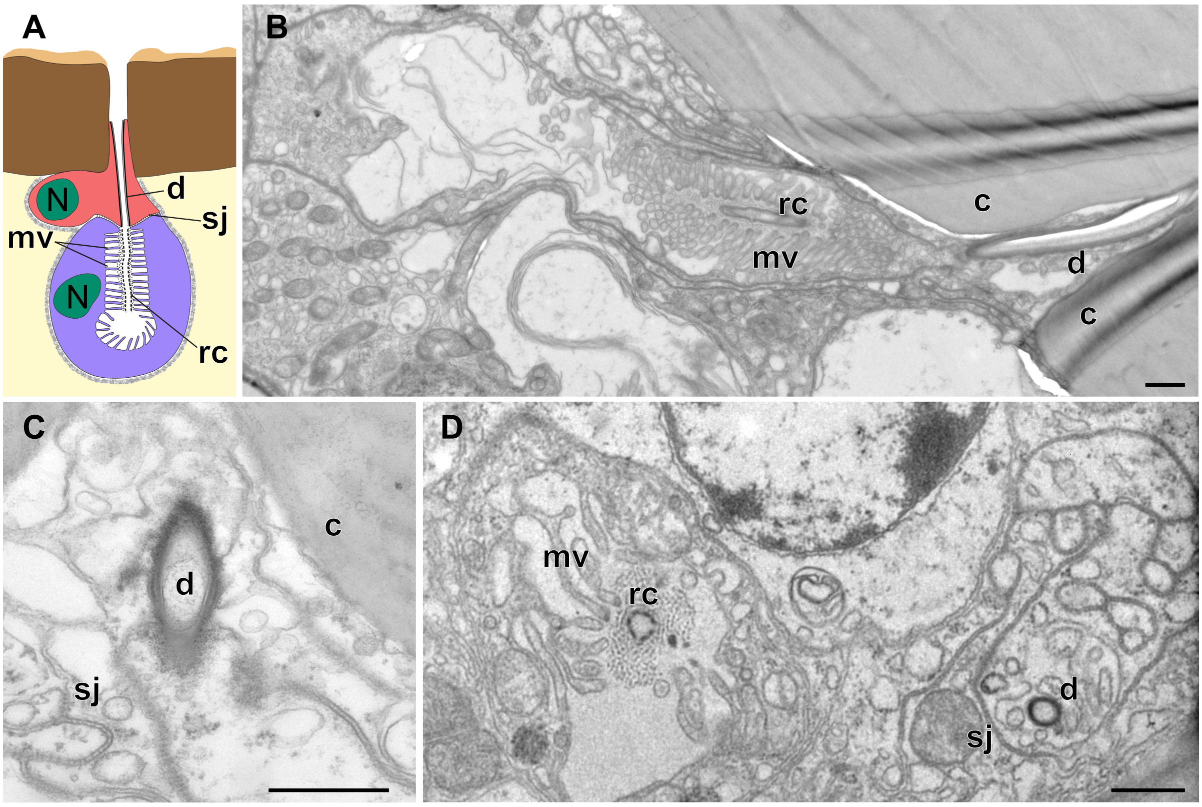

Figure S2. The bicellular exocrine glands (class 3 in the scheme of (32)) found below the propleural chest plates of the higher attine ant species *Mycetomoellerius zeteki* and the lower attine ant species *Cyphomyrmex costatus*. (**A**) Schematic drawing reproduced here from Figure 2 for convenience because the gland structure is identical for the *Mycetomoellerius*, *Paratrachymyrmex* and *Cyphomyrmex* species investigated (duct cell red, secretory cell violet, cuticle brown); (**B**) a chest plate gland of *M. zeteki*; (**C**) close up of a gland duct close to the cuticle of *M. zeteki*; (**D**) a duct and secretory gland cell from a single gland in *C. costatus* with the duct visible within the duct cell which is connected to the adjacent cell by a septate junction, while the receiving canal and the microvilli around it are visible within the secretory cell. Abbreviations refer to: nucleus (**N**), microvilli (**mv**), receiving canal (**rc**), duct (**d**), cuticle (**c**), and septate junction (**sj**) which always strengthens the contact between secretory and duct cells in a class 3 exocrine gland. Scale bars are 0.5 µm.

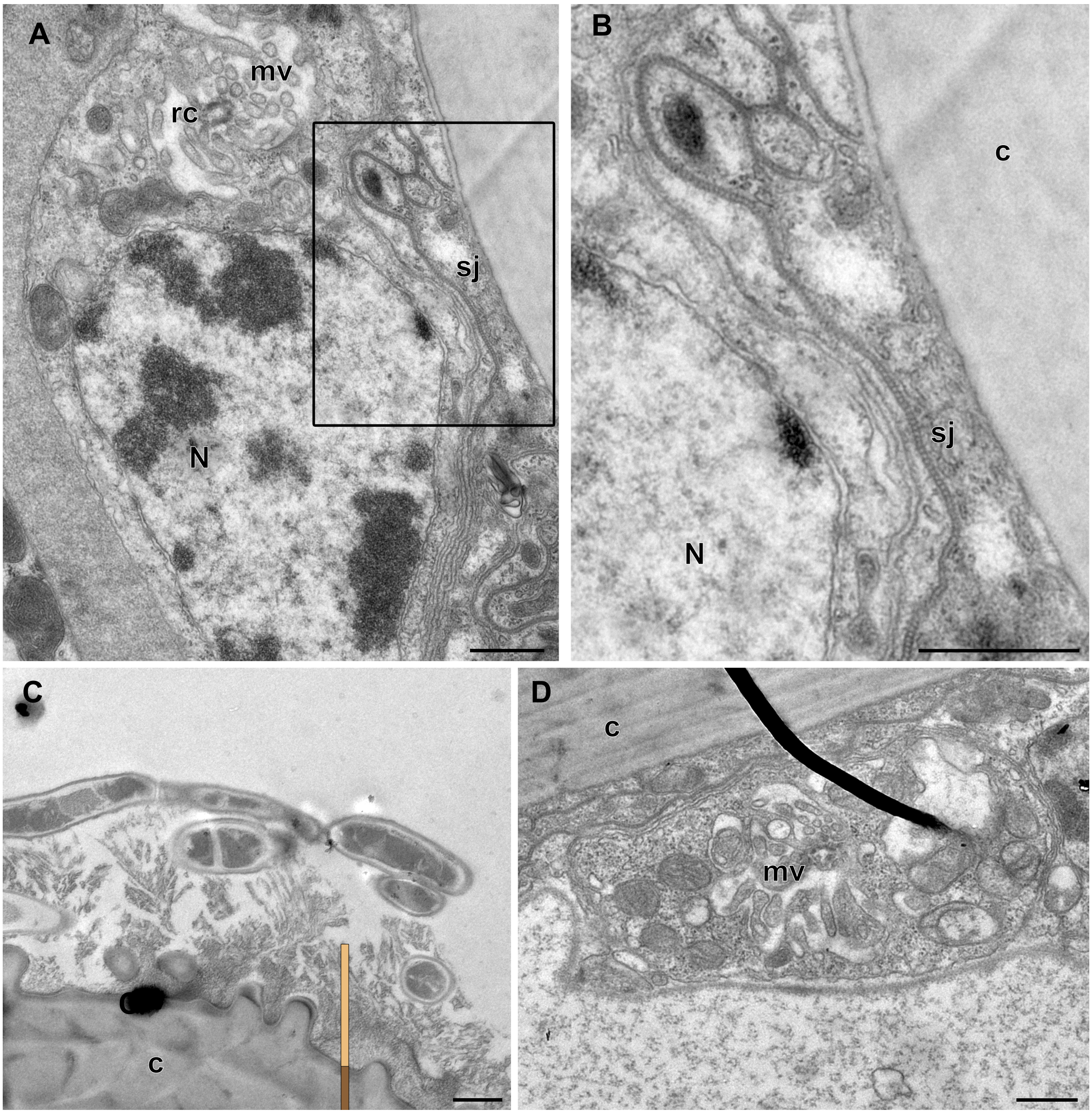

Figure S3. The exocrine glands and bacterial layer found, respectively, below and upon the propleural chest plates and mesosternum of the lower attine ant *Apterostigma dentigerum*. The morphological characteristics suggest that these glands are also typical bicellular exocrine glands (class 3), similar to the *Cyphomyrmex* and *Paratrachymyrmex* glands (Figures 2, S2) but less elaborate than in *Acromyrmex* (Figure S1). The exocrine glands had similar morphology but were found at lower density under the cuticle compared to all other attine species investigated. Images show: (**A**) A secretory gland cell; (**B**) The septate junction at higher magnification corresponding to the inserted frame in panel A; (**C**) Filamentous bacteria and secreted glandular substance on the cuticular surface (marked with a similar bicolored bar as in Figure 2); (**D**) A secretory cell under the cuticle of the mesosternum. Abbreviations indicate: nucleus (**N**), microvilli (**mv**), receiving canal (**rc**), cuticle (**c**), and the septate junction (**sj**). Scale bars are 0.5 µm.

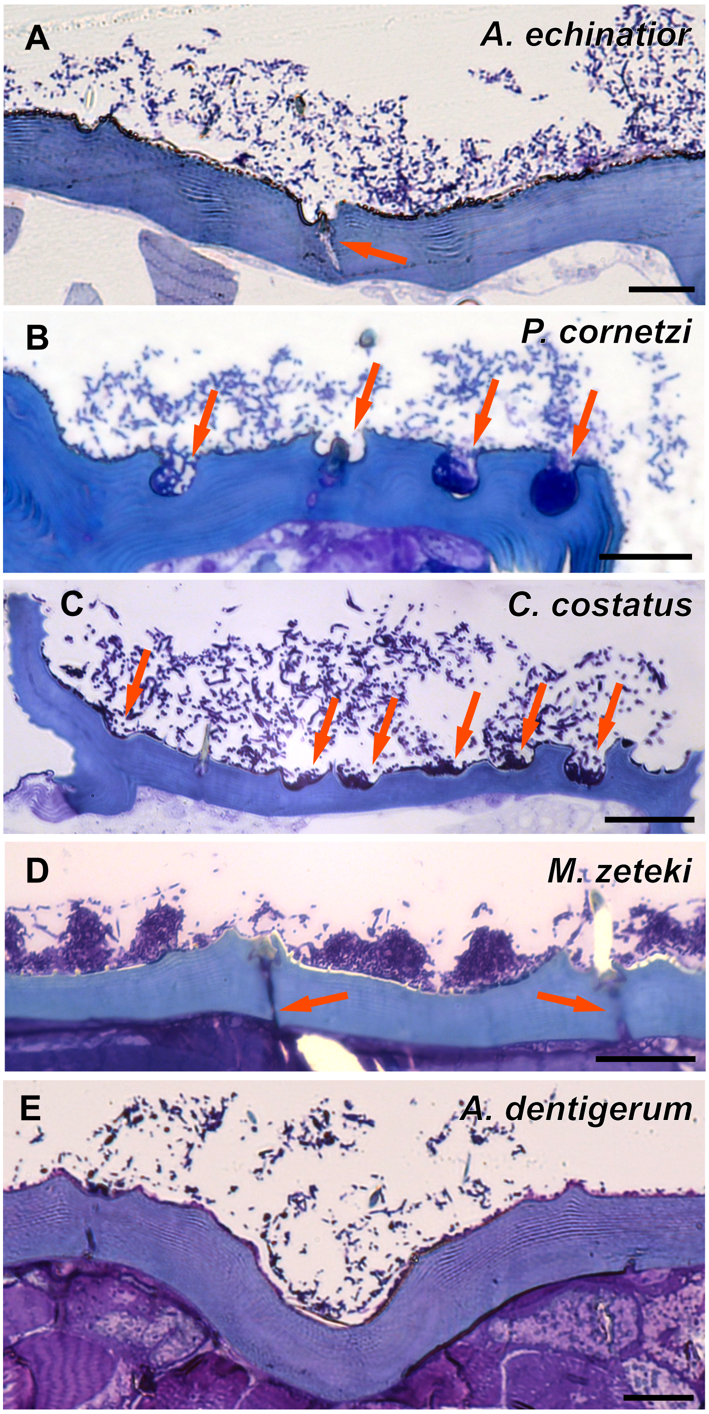

Figure S4. Light microscopy images of the cuticular surface structures and associated bacterial growth-layers in the five fungus-farming attine ant species investigated. (**A**) *A. echinatior* does not have specialized foveae (crypts) on the cuticle and bacteria thus grow evenly across the cuticular surface; (**B**) *P. cornetzi* and (**C**) *C. costatus* have similar foveae on the cuticular surface (arrowheads), each associated with a bicellular exocrine gland below the cuticle, and with bacterial growth spreading over the cuticular surface beyond these specialized structures. Measuring 15-20 foveae per ant species and comparing the largest ones (implying the sections went through the middle of the fovea), showed that the average maximal diameter of foveae is 9.4 µm in *P. cornetzi* and 8.5 µm in *C. costatus*, and that they are spaced out at mean distances of 20.6 µm (range 11.7-39.6 µm) and 10.8 µm (range 1.5-32.5 µm), respectively, in our sections of the central part of the propleural chest plates; (**D**) bacteria form an uneven growth-layer on the cuticular surface of *M. zeteki*, which does not have specialized foveae; (**E**) bacterial growth on the cuticle of *A. dentigerum* is concentrated close to the median groove along the mesosternum, but without there being foveae. Arrows in panels (A-C) indicate channels that are potentially involved in secretion of the nutritional glandular substance that supports bacterial growth. Scale bars are 20 µm.

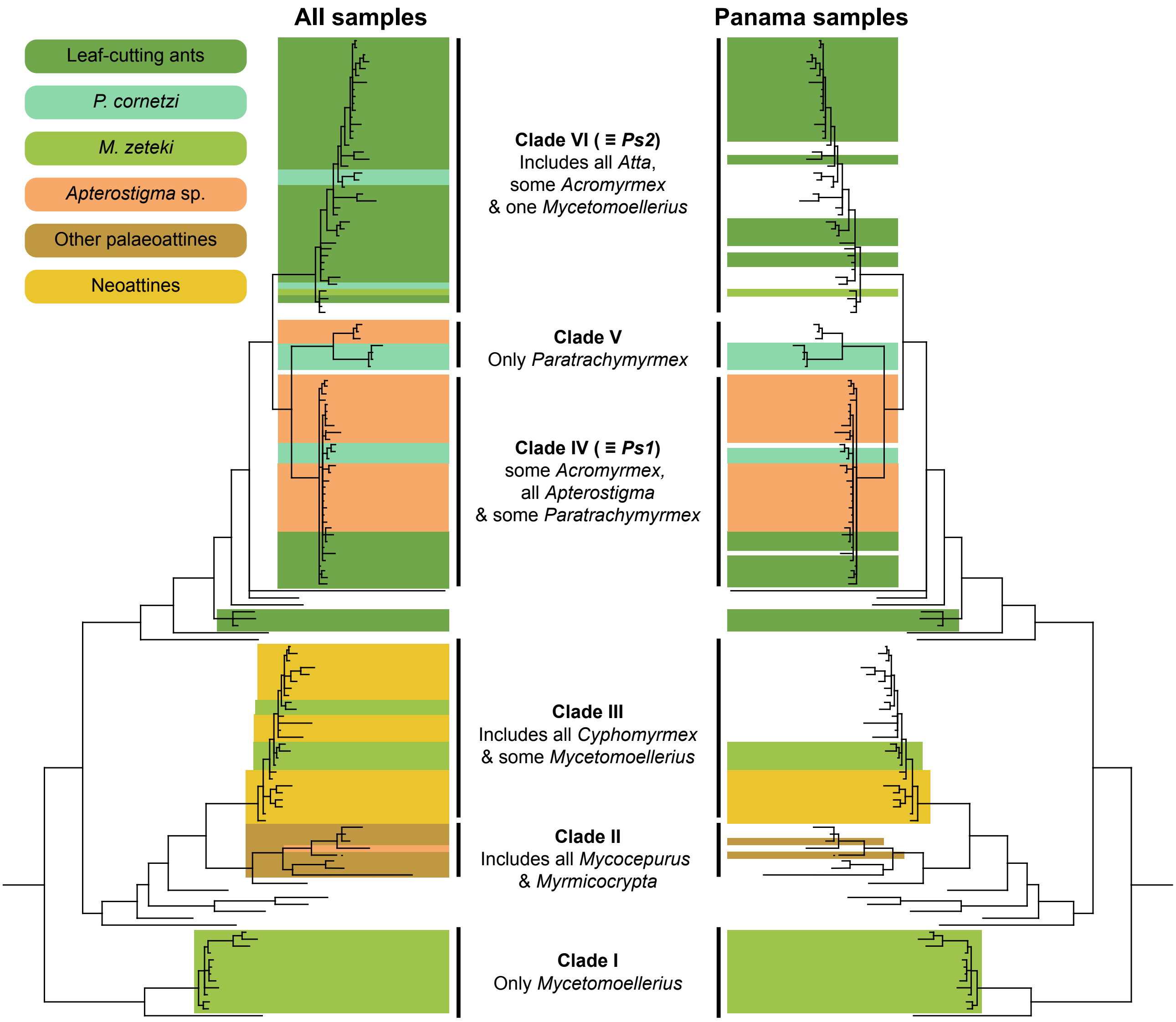

Figure S5. Phylogeny of attine-ant-associated *Pseudonocardia*, adapted from Cafaro et al. (2011). Color shading indicates the host ant species sampled in that study to identify the six *Pseudonocardia* clades (I-VI) associated with the different lineages of attine ants, using color shading that is similar to the background colors in Figure 1 to illustrate that the *Pseudonocardia* crown group lineages are associated with the later evolving (in Central/North America) crown group of the attine ants (*P. cornetzi* and *Acromyrmex*) plus phylogenetically basal *Apterostigma* (color legend). The left-hand tree reflects the attine-*Pseudonocardia* associations across 126-samples from Latin America as analyzed by (19), i.e. from sites in Central and South America, plus North America for one species. Branches falling outside the colored backgrounds are free-living *Pseudonocardia* isolates from a range of origins that were included in the phylogenetic analysis. The right-hand tree is identical, but the color shading is modified to show only the *Pseudonocardia* associations for samples used by Cafaro et al. that were collected in Panama (i.e. samples from other regions than Panama are not highlighted, so this tree is directly comparable to the results of our present study). Text in the central column provides details about the association of *Pseudonocardia* lineages with specific host ant genera or species in Panama. The trees thus depict the attine-*Pseudonocardia* associations known prior to our study (but often without having identified whether the association was cuticular or intestinal) supplemented with our attine-ant lineage-specific color shading to facilitate interpretation of our present results comparing newly obtained *Pseudonocardia* sequences of certain cuticular origin and sequences of certain intestinal origin by (7).

Comparison between the *Pseudonocardia* sequences identified as being attine-ant-associated by Cafaro et al. (19) and the *ActAcro1* representative OTU sequence originally obtained from gut microbiomes showed that essentially all sequences included in the Cafaro *et al*. study, as represented by the phylogeny in Figure S5, fall within the *ActAcro1* OTU (capturing 85 of the 87 available sequences associated with attine ants based on a 97% similarity threshold; see Table S9). We thus inferred that the cuticular *Pseudonocardia* sequences obtained in our present study form a single derived clade within the OTU

ActAcro1, in a tree that also covers the intestinal *Pseudonocardia* of the 11 Panamanian attine ant species (7; see also Table S9). The evolutionarily derived *Pseudonocardia* clade encompasses *Ps1* - identified as a good species (*P. octospinosus*) by Holmes et al. (10) and assigned as Clade IV by Cafaro et al. - and *Ps2*, likewise identified as a good species (*P. echinatior*) by Holmes et al. and assigned as Clade VI by Cafaro et al.. Furthermore, there appears to be a third actinobacterial species identified as Clade V by Cafaro et al. associated with the cuticles of *P. cornetzi* and with *Apterostigma* species elsewhere in Central/South America. The geographic patterning of associations with *Apterostigma* species seems particularly interesting, as all samples from Panamanian species rear Clade IV (*Ps1*), all Clade V symbionts stem from *Apterostigma* species sampled in Costa Rica, and South American *Apterostigma* species sampled in Ecuador were associated with the basal Clade II. These results are consistent with *Pseudonocardia* having become a cuticular symbiont of *Apterostigma* only after the attine ants had colonized Central/North America and evolved new lineages there (represented by extant *P. cornetzi* and *Acromyrmex* in this study), and with some originally South American species having secondarily acquired cuticular *Pseudonocardia* after representatives of the Central/North American lineages became sympatric with *Apterostigma* species that evolved in South America. Our Panamanian data clearly indicate that *Pseudonocardia* on the cuticle and in the gut are mutually exclusive (Figure 3), but whether this would also be true for South American *Apterostigma* remains to be seen.

As it appears, the entire crown group of the *Pseudonocardia* lineage (Clade IV, V, VI) has few known free-living species, in contrast to the more basal *Pseudonocardia* grade that is also captured within the 97% OTU *ActAcro1*. It seems likely that free-living *Pseudonocardia* are severely under-sampled, but a higher similarity between basal, gut-associated and free-living *Pseudonocardia* would be consistent with a facultative domestication scenario of ancestral *Pseudonocardia* as gut symbionts that realized only some extent of coadaptation with their ant hosts, very similar to the extent of domestication of the fungal cultivars of the paleoattine and basal neoattine (i.e. lower) attine ants that also continue to have free-living relatives (33, 34). Free-living cultivar lineages were lost when the ant cultivars evolved gongylidia to feed the ants when the attine host ants colonized dry habitats in South America via the *Mycetomoellerius* lineage (15) but our present data suggest that a similar shift to obligate highly coadapted symbiosis happened only for *Pseudonocardia* after these bacteria became cuticular symbionts nourished by specialized cuticular glands in the new Central/North American lineages (Figure 2). Both crucial shifts thus appear to have required major (geographic) vicariance events: the colonization of dry South American habitats for the cultivars and the colonization of Central/North America for *Pseudonocardia*.

For the basal part of the trees in Figure S5, Cafaro et al. (19) reported three branches associated with attine ants, one of them (Clade II) only with *Mycocepurus*, *Myrmicocrypta* and *Apterostigma* species, sometimes jointly referred to as the paleoattines (34), and another (Clade III) primarily associated with *Cyphomyrmex* species and other neoattines, the sister group to the paleoattines that later produced the *Mycetomoellerius*, *Sericomyrmex*, *Paratrachymyrmex* and *Trachymyrmex* higher attine ants and the *Atta* and *Acromyrmex* leaf-cutting ants (35; see Figure 1). As (19) noted there is one Panamanian species, *M. zeteki*, which has an exclusive association with what appears to be the most basal lineage (Clade I) of *Pseudonocardia*, while also maintaining associations with Clade III and (rarely) Clade VI *Pseudonocardia*. Whether this unusually catholic association of a single attine ant species with a variety of *Pseudonocardia* lineages is ancestral or secondarily acquired remains to be investigated by comparison across different species in the genus *Mycetomoellerius*. The only other case of exceptional diversity among *Pseudonocardia* symbionts is *A. dentigerum*, which shares Clade II *Pseudonocardia* with other Paleoattine species, but also has associations with crown group *Pseudonocardia* belonging to Clades IV and V. Here it will be of interest to establish, once more via comparative analysis across multiple *Apterostigma* species, whether the Clade II *Pseudonocardia* are always gut symbionts, and those in Clades IV and V are always cuticular symbionts of *Apterostigma* species.

3.2 Supplementary Tables

Table S1. OTU tables for (A) the rarefied Illumina MiSeq sequencing data obtained from propleural chest plates as shown in Figure 1 and (B) the unrarefied Illumina MiSeq sequencing data obtained from the same specimens. Abbreviations P and A are used for Pseudonocardiaceae and Actinobacteria (including *Pseudonocardia*), respectively. (C) a taxonomic summary of those OTUs appearing in (A) and (B).

Available as a separate file: Table S1.xlsx

Table S2. The results of generalized linear mixed models (GLMMs) with negative binomial errors, examining the OTU richness (number of different OTUs based on 97% similarity) in the cuticular microbiomes obtained from the propleural chest plates of 11 species of attine ant for all bacterial OTUs per sample in **(a)** the rarefied and **(b)** the unrarefied datasets, and the richness of OTUs assigned to Actinobacteria in **(c)** the rarefied and **(d)** the unrarefied datasets. For the main models, ant species, ant worker age (callow or forager) and their interaction were included as main effects, and colony ID was included as a random effect. Also the negative binomial dispersion parameter (θ) is given, as estimated by *glmmTMB* (13). Since there was no significant Species × Worker age interaction in any test, means and confidence intervals are given per species. Letters to the left of the values represent differences between attine host species based on Sidăk post-hoc tests, with species sharing the same letter being not significantly different in number of reads. Separate models were also constructed with reported unspecified bacterial bloom on ant host species' worker cuticles (categorized following Figure 1 as often present, occasionally present, or absent), and the area of origin of each species (South- or Central/North America – see Figure 1) as explanatory variables, in these cases using colony ID nested within species as random effects. Tests are summarized in the lower table, and those significant at P < 0.05 are marked in bold.

	**(a)** Number of rarefied bacterial OTUs per ant worker		**(b)** Number of unrarefied bacterial OTUs per ant worker		**(c)** Number of rarefied actinobacterial OTUs per ant worker		**(d)** Number of unrarefied actinobacterial OTUs per ant worker	
**Attine host**	**Mean**	**95% CI**	**Mean**	**95% CI**	**Mean**	**95% CI**	**Mean**	**95% CI**
Atta colombica	^{ab}5.26	3.35-8.25	^{abc}6.69	4.22-10.70	^{ab}0.29	0.03-2.50	^{ab}0.63	0.10-3.74
Acromyrmex echinatior	^{cd}10.28	8.33-12.55	^{de}18.54	14.88-23.10	^{b}2.10	0.85-5.21	^{b}3.31	1.34-8.16
Acromyrmex octospinosus	^{abcd}7.03	4.81-10.38	^{abc}8.85	5.81-13.33	^{ab}1.59	0.34-7.44	^{ab}1.68	0.35-7.98
Paratrachymyrmex cornetzi	^{bcd}9.97	7.03-14.15	^{cd}12.30	8.41-17.81	^{ab}1.00	0.23-4.41	^{ab}1.25	0.28-5.47
Mycetomoellerius zeteki	^{a}4.14	2.83-6.11	^{a}6.11	4.18-8.85	^{a}0.10	0.01-1.00	^{a}0.19	0.03-1.33
Sericomyrmex amabilis	^{a}4.90	3.53-6.89	^{ab}6.69	4.76-9.49	^{ab}0.00	-	^{a}0.27	0.06-1.30
Cyphomyrmex longiscapus	^{abc}5.00	2.77-8.94	^{abc}6.82	3.86-12.06	^{ab}0.55	0.07-4.14	^{ab}0.64	0.09-4.69
Cyphomyrmex costatus	^{bcd}9.21	6.55-12.94	^{abc}10.70	7.39-15.64	^{ab}0.88	0.21-3.66	^{ab}1.09	0.27-4.42
Apterostigma dentigerum	^{ab}5.58	3.90-7.92	^{abc}8.00	5.53-11.47	^{ab}0.42	0.10-1.87	^{ab}0.79	0.21-3.03
Myrmicocrypta ednælla	^{abcd}8.85	4.39-17.99	^{bcde}16.28	7.69-34.12	^{ab}1.00	0.04-25.69	^{ab}1.00	0.04-26.74
Mycocepurus smithii	^{d}12.81	9.03-18.36	^{e}27.66	19.30-40.04	^{ab}0.82	0.17-3.90	^{ab}2.01	0.48-8.39

	**(a)** Number of rarefied bacterial OTUs per ant worker				**(b)** Number of unrarefied bacterial OTUs per ant worker				**(c)** Number of rarefied actinobacterial OTUs per ant worker				**(d)** Number of unrarefied actinobacterial OTUs per ant worker			
	**df**	**Wald χ^2**	**P**	θ	**df**	**Wald χ^2**	**P**	θ	**df**	**Wald χ^2**	**P**	θ	**df**	**Wald χ^2**	**P**	θ
Species	**10**	**96.963**	**<0.0001**		**10**	**170.644**	**<0.0001**		**10**	**27.561**	**0.0163**		**10**	**28.500**	**0.0015**	7.05
Worker age	1	0.001	0.9744	10.39	1	0.188	0.6647	9.57	1	6.512	0.2596	80.19	1	1.498	0.2210	
Species × Worker age	10	12.486	0.2538		10	10.822	0.3716		10	7.011	0.7244		10	8.179	0.6114	
Colony (Random)	1	0.374	0.5410		1	3.742	0.0531		1	0	1*		1	0	1*	
Visible bloom	2	2.844	0.2413	9.07	2	4.507	0.1050	8.70	2	2.569	0.2768	58.95	2	1.2348	0.5393	5.40
Origin	1	0.456	0.4995	9.07	1	0.364	0.5463	8.70	1	0.092	0.7617	66.02	1	0.0535	0.8171	5.40

*This is a sanity check. The random effect should always be zero and with p = 1 for the overall OTU richness of the rarefied dataset

Table S3. Summary of rarefied data presented in Figure 1 and additional data on the relative abundance of sequences within all actinobacterial OTUs identified. (A) The number of colonies sampled per attine species, ranked after their position in the phylogeny, and the mean proportional abundance of sequences assigned to actinobacterial OTUs, or to the *Pseudonocardia* OTU, relative to the total of all identified OTUs in the cuticular microbiomes. Proportions are given separately for newly hatched callow workers and older foraging workers, as earlier studies have suggested that microbiome diversity increases with worker age so that vertically acquired *Pseudonocardia* are more dominant in the cuticular microbiomes of younger *Acromyrmex* workers (3, see also 11), a contention that is supported by the present data. *Pseudonocardia* abundances represent all sequences classified within a single OTU at 97% sequence similarity, equivalent to the *ActAcro1* OTU identified by Sapountzis et al. (7) for gut-associated *Pseudonocardia*, which is known to include the two known *Acromyrmex*-associated *Pseudonocardia* species *P. octospinosus* and *P. echinatior* (10). (B) The mean percentage representation of sequences within all 15 identified families (18 identified genera) of Actinobacteria present across the sampled cuticular microbiomes, with the number of OTUs per genus in brackets. *Pseudonocardia* are marked as bold, and unclassified/uncultured taxa in shades of grey. Cells are colored in shades of red according to the values they contain. All numbers are based on rarefied data. Comparison with the unrarefied data Table showed that an additional two orders, six families and seven identified genera of bacteria were removed by rarefaction, which were present in negligible numbers (<0.01%) of reads. These were representatives of the genera *Conexibacter* (Solirubrobacterales), *Adlercreutzia* (Coriobacteriales), *Brachybacterium*, *Micrococcus*, *Micromonospora*, *Williamsia*, *Propioniferax* (all Actinomycetales) and an unidentified member of the AKIW543 group of the Rubrobacteridae (Table S1c).

A

Ant species	Phylogenetic position	Number of colonies sampled	Callow workers: Mean proportion of sequences within actinobacterial OTUs relative to total number of sequenced bacterial OTUs (±SE)	Callow workers: Mean proportion of sequences within the *Pseudonocardia* OTU relative to total number of sequenced bacterial OTUs (±SE)	Callow workers: Mean proportion of sequences within the *Pseudonocardia* OTU relative to total number of sequenced actinobacterial OTUs (±SE)	Foraging workers: Mean proportion of sequences within actinobacterial OTUs relative to total number of sequenced bacterial OTUs (±SE)	Foraging workers: Mean proportion of sequences within the *Pseudonocardia* OTU relative to total number of sequenced bacterial OTUs (±SE)	Foraging workers: Mean proportion of sequences within the *Pseudonocardia* OTU relative to total number of sequenced actinobacterial OTUs (±SE)
Atta colombica	Leaf-cutting	4	0.037 ± 0.030	0 ± 0	0 ± 0	0.011 ± 0.011	0 ± 0	0 ± 0
Acromyrmex echinatior	Leaf-cutting	11	0.128 ± 0.038	0.093 ± 0.029	0.588 ± 0.094	0.147 ± 0.036	0.095 ± 0.034	0.575 ± 0.092
Acromyrmex octospinosus	Leaf-cutting	4	0.101 ± 0.067	0.100 ± 0.067	0.836 ± 0.140	0.166 ± 0.049	0.019 ± 0.090	0.175 ± 0.082
Paratrachymyrmex cornetzi	Higher attine	5	0.140 ± 0.058	0.077 ± 0.050	0.524 ± 0.164	0.016 ± 0.016	0.010 ± 0.010	0.375 ± 0.183
Mycetomoellerius zeteki	Higher attine	8	0 ± 0	0 ± 0	0 ± 0	0.052 ± 0.052	0 ± 0	0 ± 0
Sericomyrmex amabilis	Higher attine	7	0.003 ± 0.002	0 ± 0	0 ± 0	0 ± 0	0 ± 0	0 ± 0
Cyphomyrmex longiscapus	Lower attine	4	0.074 ± 0.074	0 ± 0	0 ± 0	0.144 ± 0.143	0 ± 0	0 ± 0
Cyphomyrmex costatus	Lower attine	6	0.065 ± 0.049	0.00007 ± 0.00007	0.003 ± 0.003	0.150 ± 0.105	0.00006 ± 0.00006	0.001 ± 0.001
Apterostigma dentigerum	Lower attine	7	0.139 ± 0.136	0.0001 ± 0.0001	0.143 ± 0.143	0.004 ± 0.003	0.00014 ± 0.0001	0.071 ± 0.071
Mycocepurus smithii	Lower attine	5	0.076 ± 0.037	0 ± 0	0 ± 0	0.009 ± 0.006	0 ± 0	0.000 ± 0.045
Myrmicocrypta ednaella	Lower attine	1	0 ± 0	0 ± 0	0 ± 0	0.004 ± 0.002	0.00025 ± 0.00025	0.045 ± 0.000

Table S3: *Continued*

B

			Atta colombica		*Acromyrmex echinatior*		*Acromyrmex octospinosus*		*Paratrachymyrmex cornetzi*		*Mycetomoellerius zeteki*		*Sericomyrmex amabilis*		*Cyphomyrmex longiscapus*		*Cyphomyrmex costatus*		*Apterostigma dentigerum*		*Myrmicocrypta ednaella*		*Mycocepurus smithii*	
**Order**	**Family**	**Genus/Group**	**C**	**F**	**C**	**F**	**C**	**F**	**C**	**F**	**C**	**F**	**C**	**F**	**C**	**F**	**C**	**F**	**C**	**F**	**C**	**F**	**C**	**F**
Actinomycetales	Actinomycetaceae	Actinomyces (2)	0	0	0	0.06	0	0.02	0	0	0	0	0	0	0.04	0	0	0	0	0	0	0	0	0
	Cellulomonadaceae	*Actinotalea*	0	0	0.99	1.33	0	0.89	0	0	0	0	0	0	0	0	0	0	0	0	0	0	0	0
		Cellulomonas	0	0	0	0	0	0	0	0	0	0	0	0	0	0	0	0	0	0	0	0	0.46	0
	Corynebacteriaceae	*Corynebacterium* (4)	0.01	0	0	0.41	0	0	4.66	0	0	5.23	0	0	6.13	14.4	0	3.15	0	0	0	0.25	0	0.13
		unclassified	3.03	0	0.07	0	0	0.03	0	<0.01	0.01	0	0.13	0	1.24	0	5.14	0	0.16	0.22	0.03	0.05	0	0.01
	Intrasporangiaceae	unclassified (2)	0	0	0.59	0.68	0	0	0	0	0	0	0	0	0	0	0	0	0	0	0	0	0	0
	Kineosporiaceae	*Kineosporia*	0	0	0	0	0	0	0	0	0	0	0	0	0	0	0	0	13.7	0	0	0	0	0
	Microbacteriaceae	Candidat. *Rhodoluna*	0	0	0	<0.01	0.06	1.53	0.74	0.43	0	0	0.13	0	0	0.02	1.19	0.29	0.01	0.01	0	0	0.58	0.61
		Leifsonia	0	0	0.01	0.11	0	0.01	0	<0.01	0	0	0	0	0	0	0	0	0	0	0	0	0	0
		uncultured	0	0	0	0	0	0	<0.01	0.14	0	0	0	0	0	0	0	0.24	0	0	0	0	0.03	0
	Micrococcaceae	*Arthrobacter*	0	0	0	0.89	0	0	0	0	0	0	0	0	0	0	0	0	0	0	0	0	0	0
		Kocuria	0	0	0.30	0	0	0	0	0	0	0	0	0	0	0	0	<0.01	0	0	0	0	0	0
	Nakamurellaceae	Saxeibacter	0	0	0.03	0.06	0	0	0	0	0	0	0	0	0	0	0	0	0	0	0	0	0	0
	Nocardiaceae	*Nocardia*	0	0	0	0	0	0	0	0	0	0	0	0	0	0	0	0	0	0.15	0	0	0	0
	Nocardioidaceae	*Aeromicrobium*	0	0	0.51	0.66	0	8.94	0	0	0	0	0	0	0	0	0	0	0	0	0	0.03	0	0
		Marmoricola	0	0	0	0	0	0	0	0	0	0	0	0	0	0	0	0	0	0	0	0	0	0.02
		Nocardioides	0	0	0	0.01	0	0	0	0	0	0	0	0	0	0	0	0	0	0	0	0	0	0
		Nocardiopsis	0	0	0	0	0	0	0	0	0	0	0	0	0	0	0	0	0	0	0	0	0.99	0
	Propionibacteriaceae	*Propionibacterium*	0	0	0.03	<0.01	0	<0.01	0	0	0	0	0	0	0	0	0	0	<0.01	<0.01	0	0	0	0
	Pseudonocardiaceae	***Pseudonocardia***	**0**	**0**	**930**	**9.52**	**10.0**	**1.93**	**7.73**	**1.01**	**0**	**0**	**0**	**0**	**0**	**0**	**0.01**	**0.01**	**0.01**	**0.01**	**0**	**0.03**	**0**	**0**
		unclassified	0	0	<0.01	0.87	0	0	0	0	0	0	0	0	0	0	0	0	0	0	0	0	0	0
	Sporichthyaceae	hgcl_clade	0.02	1.06	0	0	0	0	0.88	<0.01	0	0	0	0	0	0	0.11	11.3	0	0	0	0	1.47	0.05
	unclassified	unclassified	0	0	0.05	0.04	<0.01	3.23	0	0	0	0	0	0	0	0	0	0	0	0	0	0	0	0
Bifidobacteriales	Bifidobacteriaceae	*Bifidobacterium*	0	0	0	0.06	0	0	0	0	0	0	0	0	0	0	0	0	0	0	0	0	0	0
	Gardnerella	unclassified	0	0	0	0	0	0	0	0	0	0	0	0	0	0	0	0	0	0	0	0	0	0.12
Coriobacteriales	Coriobacteriaceae	*Olsenella*	<0.01	0.19	<0.01	0.89	0	0	0	0	0	0	<0.01	0	0	0	0	0	0	0	0	0	<0.01	9.76

Table S4. The results of generalized linear mixed models (GLMMs) with negative binomial errors, examining (A) the number of bacterial OTU reads assigned to Actinobacteria, and (B) the total number of bacterial OTU reads assigned to *Pseudonocardia* per sample in the rarefied dataset. For the main models, ant species, ant worker age (callow or forager) were included as main effects, and colony ID was included as a random effect. The negative binomial dispersion parameter (θ) is also given, as estimated by *glmmTMB* (13). The sparse nature of the rarefied dataset meant that the interaction between ant species and worker age could not be fitted (Of all possible combinations, 46% had zero Actinobacteria and 70% had zero *Pseudonocardia* reads). Letters to the left of the values represent differences between attine host species and worker ages based on Sidăk post-hoc tests, with species sharing the same letter being not significantly different in number of reads. Separate models were also constructed with reported bacterial bloom on ant host species' worker cuticles (categorized following Figure 1 as often present, occasionally present, or absent), and the area of origin of each species (South- or Central/North America – see Figure 1) as explanatory variables, using colony ID nested within species as random effects. Tests are summarized in the lower table, and those significant at P < 0.05 are marked in bold.

	(A) Number of Actinobacteria reads			(B) Number of *Pseudonocardia* reads		
**Attine host**		**Mean**	**95% CI**		**Mean**	**95% CI**
Atta colombica	abc	18.3	0.438-765	abcd	0.00	-
Acromyrmex echinatior	c	382	54.7-2670	cd	1260	245-6000
Acromyrmex octospinosus	c	393	15.3-10100	cd	994	60.3-16300
Paratrachymyrmex cornetzi	bc	128	4.87-3360	cd	255	18.2-3290
Mycetomoellerius zeteki	ab	0.759	0.017-33.8	abcd	0.00	-
Sericomyrmex amabilis	a	0.351	0.0117-10.6	a	0.08	0.00-4.48
Cyphomyrmex longiscapus	abc	99.9	1.18-8430	abcd	0.00	-
Cyphomyrmex costatus	bc	131	4.42-3900	ab	0.13	0.00-9.03
Apterostigma dentigerum	abc	6.75	0.313-145	ab	0.25	0.01-9.97
Myrmicocrypta ednælla	abc	7.19	0.0115-4490	abcd	0.00	-
Mycocepurus smithii	abc	18.4	0.592-572	ab	0.16	0.00-16.4

	Number of Actinobacteria reads				Number of *Pseudonocardia* reads			
	**df**	**χ^2**	**P**	θ	**df**	**χ^2**	**P**	θ
Species	**10**	**41.48**	**<0.0001**		**10**	**340.423**	**<0.0001**	
Worker age	1	0.85	0.3565	0.202	1	0.006	0.9396	0.227
Colony (Random)	1	0	1		1	0	1	
Visible bloom	**2**	**16.053**	**0.0003**	0.134	2	4.266	0.1185	0.201
Origin	1	0.039	0.8435	0.166	1	0.236	0.6268	0.201

*The sparse data in the for *Pseudonocardia* abundance meant that the interaction between ant species and worker age could not be fitted (36% of all possible combinations consistently had zero *Pseudonocardia* reads).

Table S5. The results of generalized linear mixed models (GLMMs) with beta-binomial errors, examining the proportion of all bacterial OTU reads that were classified as Actinobacteria and the proportion of all actinobacterial reads that were classified as *Pseudonocardia*, based on the unrarefied dataset. For the main models, ant species, ant worker age (callow or forager) and their interaction were included as main effects, and colony ID was included as a random effect. The beta-binomial dispersion parameter (θ) is given, as estimated by *glmmTMB* (13). Separate models were also constructed with reported unspecified bacterial bloom on ant host species' worker cuticles (categorized following Figure 1 as often present, occasionally present, or absent), and the area of origin of each species (South- or Central/North America – see Figure 1) as explanatory variables, using colony ID nested within species as random effects. Tests are summarized in the lower table, and those significant at P < 0.05 are marked in bold.

	Proportion of all bacterial OTUs that are Actinobacteria			Proportion of actinobacterial OTUs that are *Pseudonocardia*		
**Attine host**		**Mean**	**95% CI**		**Mean**	**95% CI**
Atta colombica	ab	0.014	0.003-0.059	ab	0	-
Acromyrmex echinatior	d	0.107	0.064-0.177	b	0.274	0.139-0.468
Acromyrmex octospinosus	cd	0.097	0.042-0.211	ab	0.202	0.055-0.524
Paratrachymyrmex cornetzi	bcd	0.049	0.018-0.125	ab	0.278	0.079-0.631
Mycetomoellerius zeteki	ab	0.006	0.001-0.035	ab	0	-
Sericomyrmex amabilis	a	0.008	0.002-0.029	ab	0.020	0.001-0.361
Cyphomyrmex longiscapus	abcd	0.023	0.005-0.103	ab	0	-
Cyphomyrmex costatus	abcd	0.041	0.016-0.104	a	0.030	0.004-0.178
Apterostigma dentigerum	abc	0.024	0.009-0.063	ab	0.065	0.012-0.284
Myrmicocrypta ednaella	abcd	0.026	0.004-0.161	ab	0.041	0.001-0.661
Mycocepurus smithii	abcd	0.047	0.017-0.121	a	0.017	0.002-0.138

	Proportion of all bacterial OTUs that are Actinobacteria				Proportion of actinobacterial OTUs that are *Pseudonocardia*			
	**df**	**Wald χ^2**	**P**	**θ**	**df**	**Wald χ^2**	**P**	**θ**
Species	**10**	**70.739**	**<0.0001**	4.26	**10**	**30.930**	**0.0006**	2.92
Worker age	1	0.139	0.7091		1	0.045	0.8317	
Species × Worker age	10	16.599	0.0837			-*	-*	
Colony (Random)	1	0.491	0.4835		**1**	**10.510**	**0.0012**	
Visible bloom	**2**	**17.494**	**0.0002**	3.86	**2**	**11.602**	**0.0030**	2.92
Origin	**1**	**6.229**	**0.0126**	3.94	**1**	**7.346**	**0.0067**	2.89

*Several samples had no Actinobacteria, so calculating a proportion was impossible for some combinations of ant species and worker age, which meant the interaction could not be fitted.

Table S6. OTU tables for the rarefied Illumina MiSeq sequencing dataset obtained in parallel with the microscopy samples (TEM) to inform interpretation of the microscopy images as shown in Figures 2, S1-S3.

Available as a separate file: Table S6.xlsx

Table S7. Comparison of proportional abundances (Mean ± SE) of *Pseudonocardia* within the entire cuticular microbiome (calculated from the number of sequences within the *Pseudonocardia* OTU *ActAcro1*, relative to the total number of bacterial sequences in a sample) between worker ants sampled in the field (Figure 1) and worker ants subsequently sampled from lab colonies (or new field collections in the case of *Apterostigma dentigerum*) in parallel to sampling for TEM microscopy (Figures 2, S1-S3) for five representative attine ant species. Across the four species that were sampled in both field and lab, lab colonies had markedly higher abundances of *Pseudonocardia* on the worker cuticles, consistent with previous studies sampling workers of both origins (cuticular microbiome (3); gut microbiome (7)). Data for *A. dentigerum* suggest that *Pseudonocardia* may be more abundant, at least some of the time, on the mesosternum* than on the propleural chest plates.

Attine ant host	Proportional abundance of *Pseudonocardia* in the cuticular microbiome of workers sampled in the field	Proportional abundance of *Pseudonocardia* in the cuticular microbiome of workers sampled from lab colonies	Time between colony collection and sampling in the lab	Proportional abundance of *Pseudonocardia* in the cuticular microbiome of workers sampled from later field collections**	Time between colony collection and sampling**
Acromyrmex echinatior	0.093 ± 0.03	0.73 ± 0.09	36 months	-	-
Paratrachymyrmex cornetzi	0.077 ± 0.05	0.65 ± 0.08	12 months	-	-
Mycetomoellerius zeteki	0 ± 0	0.46 ± 0.16	36 months	-	-
Cyphomyrmex costatus	trace (<0.0001)	0.57 ± 0.11	36 months	-	-
Apterostigma dentigerum on propleural chest plate*	trace (<0.0001)	-	-	0.28 ± 0.09	newly arrived (ca. 2 weeks)
Apterostigma dentigerum on mesosternum*	trace (<0.0001)	-	-	0.77 ± 0.04	newly arrived (ca. 2 weeks)

* Reports from previous studies cite *Pseudonocardia* as being less specifically localized on propleural chest plates as is the case in other attine ant species because blooms can also be observed on the mesosternum in addition to or instead of the chest plates; we therefore sampled both cuticular areas separately (1, 2; see also, 27).

** There were no surviving *Apterostigma dentigerum* lab colonies from original field collections at the time of sampling, so new field collections were used instead for microscopy and parallel microbiome sequencing. It is interesting to note that *Pseudonocardia* abundance was considerably higher in these later-year field samples than in our original dataset (Figure 1), consistent with previously reported variability in the association between Actinobacteria and *Apterostigma* species (1, 2).

Table S8. Comparison of the mean proportional *Pseudonocardia* abundances in the cuticular and gut microbiomes of 11 attine ant species, ranked by phylogenetic position, as combined and presented in Figure 3. We list the mean proportional abundances of all sequences that were assigned to actinobacterial OTUs and the mean proportion of *Pseudonocardia* sequences as a fraction of all actinobacterial sequences, both for the cuticular microbiome and for the gut microbiome. *Pseudonocardia* abundances refer to the single 97% identity OTU corresponding to the gut microbial OTU *ActAcro1*. Cuticular microbiome data are from the present study (Figure 1) and gut microbiome data from (7).

		**Cuticular microbiome**				**Gut microbiome**			
Ant species	Phylogenetic position	Number of colonies sampled for cuticular microbiomes (this study)	*Pseudonocardia* OTUs / all OTUs in the cuticular microbiome[1] (±SE)	Actinobacterial OTUs / all OTUs in the cuticular microbiome (±SE)	*Pseudonocardia* OTUs / all actinobacterial OTUs in the cuticular microbiome (±SE)	Number of colonies sampled for abdominal microbiomes (7)	*Pseudonocardia* OTUs / all bacterial OTUs in the abdominal microbiomes[1] (±SE)	Actinobacterial OTUs / OTUs in the abdominal microbiome (±SE)	*Pseudonocardia* OTUs / all actinobacterial OTUs in the abdominal microbiome (±SE)
Atta colombica	Leaf-cutting	4	0.000 ±0	0.025 ±0.017	0 ±0	4	0.004 ±0.001	0.004 ±0.003	0.29 ±0.15
Acromyrmex echinatior	Leaf-cutting	11	0.094 ±0.003	0.14 ±0.026	0.58 ±0.06	6	0.001 ±0.0001	0.0016 ±0.0005	0.24 ±0.10
Acromyrmex octospinosus	Leaf-cutting	4	0.057 ±0.008	0.14 ±0.04	0.48 ±0.12	4	0.001 ±0.0001	0.0006 ±0.0004	0.41 ±0.17
Paratrachymyrmex cornetzi	Higher attine	5	0.046 ±0.007	0.08 ±0.03	0.45 ±0.12	4	0.000 ±0.00005	0.0005 ±0.0002	0.57 ±0.17
Mycetomoellerius zeteki	Higher attine	8	0.000 ±0	0.03 ±0.03	0 ±0	5	0.017 ±0.005	0.047 ±0.04	0.58 ±0.12
Sericomyrmex amabilis	Higher attine	7	0.000 ±0	0.001 ±0.0008	0 ±0	4	0.004 ±0.001	0.057 ±0.048	0.037 ±0.02
Cyphomyrmex longiscapus	Lower attine	4	0.000±0	0.10 ±0.07	0 ±0	3	0.002 ±0.0005	0.081 ±0.01	0.028 ±0.02
Cyphomyrmex costatus	Lower attine	6	0.000 ±0.00001	0.11 ±0.06	0.002 ±0.001	4	0.365 ±0.03	0.43 ±0.12	0.89 ±0.03
Apterostigma dentigerum	Lower attine	7	0.000 ±0.00002	0.049 ±0.045	0.095 ±0.66	4	0.064 ±0.016	0.12 ±0.06	0.47 ±0.16
Mycocepurus smithii	Lower attine	5	0.000 ±0	0.0018 ±0.001	0 ±0	2	0.179 ±0.04	0.30 ±0.06	0.45 ±0.08
Myrmicocrypta ednaella	Lower attine	1	0.000 ±0.0006	0.033 ±0.015	0.02 ±0.02	2	0.128 ±0.014	0.41 ±0.12	0.24 ±0.12

[1] These data are plotted in Figure 3

Table S9: Percentage similarity between the 87 available attine-associated cuticular *Pseudonocardia* sequences of the phylogeny from Cafaro *et al.* (19), plus 76 additional sequences obtained from GenBank, and the representative sequence for the *ActAcro1 Pseudonocardia* OTU (encompassing 404 unique sequences from the present study of cuticular microbiomes and 1095 unique sequences obtained by (7) for gut microbiomes). The cases where similarity to the *ActAcro1* representative sequence was <97% are marked in red. The E-value (Expect value) gives an estimate of the chance of sequences being identical to the *ActAcro1* OTU by chance. Colors are used to distinguish between the different *Pseudonocardia* clades identified or inferred by Cafaro *et al.* (19)

Taxon	Cafaro sample No.	Ant host species	Cafaro Clade (1-6)	Accession number	Matched sequence	% similarity with *ActAcro1*	E-value
Pseudonocardia sp.	116	*Mycetomoellerius zeteki*	1	AY376893.1	*ActAcro1*	97.24	3×10^{-122}
Pseudonocardia sp.	108	*Mycetomoellerius zeteki*	1	EU139573.2	*ActAcro1*	97.64	5×10^{-124}
Pseudonocardia sp.	126	*Mycetomoellerius zeteki*	1	EU283919.2	*ActAcro1*	96.85	1×10^{-120}
Pseudonocardia sp.	122	*Mycetomoellerius zeteki*	1	EU283930.1	*ActAcro1*	97.24	3×10^{-122}
Pseudonocardia sp.	112	*Mycetomoellerius zeteki*	1	EU283940.1	*ActAcro1*	97.24	3×10^{-122}
Pseudonocardia sp.	110	*Mycetomoellerius zeteki*	1	EU929022.1	*ActAcro1*	97.24	3×10^{-122}
Pseudonocardia sp.	113	*Mycetomoellerius zeteki*	1	EU929024.1	*ActAcro1*	97.24	3×10^{-122}
Pseudonocardia sp.	114	*Mycetomoellerius zeteki*	1	EU929025.1	*ActAcro1*	97.24	3×10^{-122}
Pseudonocardia sp.	115	*Mycetomoellerius zeteki*	1	EU929026.1	*ActAcro1*	97.24	3×10^{-122}
Pseudonocardia sp.	118	*Mycetomoellerius zeteki*	1	EU929027.1	*ActAcro1*	97.24	3×10^{-122}
Pseudonocardia sp.	123	*Mycetomoellerius zeteki*	1	EU929029.1	*ActAcro1*	97.24	3×10^{-122}
Pseudonocardia sp.	77	*Cyphomyrmex muelleri*	2	EU139569.2	*ActAcro1*	98.02	3×10^{-125}
Pseudonocardia sp.	73	*Cyphomyrmex longiscapus*	2	EU139570.2	*ActAcro1*	98.02	3×10^{-125}
Pseudonocardia sp.	72	*Cyphomyrmex costatus*	2	EU139571.2	*ActAcro1*	99.21	3×10^{-130}
Pseudonocardia sp.	93	*Mycocepurus sp.*	2	EU283917.1	Actin14	99.21	1×10^{-130}
Pseudonocardia sp.	78	*Cyphomyrmex muelleri*	2	EU283922.1	*ActAcro1*	98.02	4×10^{-125}
Pseudonocardia sp.	74	*Cyphomyrmex longiscapus*	2	EU283933.1	*ActAcro1*	98.02	3×10^{-125}
Pseudonocardia sp.	91	*Mycocepurus smithii*	2	EU283934.1	Actin14	98.43	1×10^{-126}
Pseudonocardia sp.	62	*Apterostigma dentigerum*	2	EU283937.1	Actin14	99.21	9×10^{-131}
Pseudonocardia sp.	94	*Myrmicocrypta ednaella*	2	EU283938.1	*ActAcro1*	98.02	6×10^{-125}
Pseudonocardia sp.	76	*Cyphomyrmex longiscapus*	2	EU929004.1	*ActAcro1*	98.02	6×10^{-125}
Pseudonocardia sp.	75	*Cyphomyrmex longiscapus*	2	EU929005.1	*ActAcro1*	98.02	5×10^{-125}
Pseudonocardia sp.	79	*Cyphomyrmex sp.*	2	EU929006.1	*ActAcro1*	98.02	5×10^{-125}
Pseudonocardia sp.	96	*Myrmicocrypta sp.*	2	EU929017.1	Actin14	99.21	2×10^{-130}
Pseudonocardia sp.	117	*Mycetomoellerius zeteki*	3	EF588213.1	*ActAcro1*	97.63	2×10^{-123}
Pseudonocardia sp.	121	*Mycetomoellerius zeteki*	3	EF588226.1	*ActAcro1*	98.02	5×10^{-125}
Pseudonocardia sp.	124	*Mycetomoellerius zeteki*	3	EF588230.1	*ActAcro1*	98.02	5×10^{-125}
Pseudonocardia sp.	119	*Mycetomoellerius zeteki*	3	EU283939.1	*ActAcro1*	98.02	5×10^{-125}
Pseudonocardia sp.	111	*Mycetomoellerius zeteki*	3	EU929023.1	*ActAcro1*	98.03	1×10^{-125}
Pseudonocardia sp.	120	*Mycetomoellerius zeteki*	3	EU929028.1	*ActAcro1*	98.02	5×10^{-125}
Pseudonocardia sp.	49	*Apterostigma dentigerum*	4	AY376891.1	*ActAcro1*	99.61	3×10^{-132}
Pseudonocardia sp.	50	*Apterostigma dentigerum*	4	AY376892.1	*ActAcro1*	96.56	4×10^{-121}
Pseudonocardia sp.	7	*Acromyrmex echinatior*	4	EF588211.1	*ActAcro1*	100	7×10^{-134}
Pseudonocardia sp.	70	*Atta sp.*	4	EF588215.1	*ActAcro1*	100	6×10^{-134}
Pseudonocardia sp.	29	*Acromyrmex echinatior*	4	EF588220.1	*ActAcro1*	100	7×10^{-134}
Pseudonocardia sp.	31	*Acromyrmex octospinosus*	4	EF588224.2	*ActAcro1*	100	7×10^{-134}
Pseudonocardia sp.	104	*Paratrachymyrmex cornetzi*	4	EF588227.1	*ActAcro1*	100	7×10^{-134}
Pseudonocardia sp.	99	*Paratrachymyrmex cornetzi*	4	EF588228.1	*ActAcro1*	100	5×10^{-134}
Pseudonocardia sp.	30	*Acromyrmex octospinosus*	4	EF588232.1	*ActAcro1*	100	6×10^{-134}
Pseudonocardia sp.	45	*Apterostigma dentigerum*	4	EU139566.2	*ActAcro1*	100	5×10^{-134}
Pseudonocardia sp.	46	*Apterostigma dentigerum*	4	EU139567.2	*ActAcro1*	99.61	2×10^{-132}
Pseudonocardia sp.	47	*Apterostigma dentigerum*	4	EU139568.2	*ActAcro1*	100	5×10^{-134}
Pseudonocardia sp.	107	*Paratrachymyrmex cornetzi*	4	EU283921.1	*ActAcro1*	100	5×10^{-134}
Pseudonocardia sp.	53	*Apterostigma dentigerum*	4	EU283926.1	*ActAcro1*	100	7×10^{-134}
Pseudonocardia sp.	48	*Apterostigma dentigerum*	4	EU283928.1	*ActAcro1*	100	5×10^{-134}
Pseudonocardia sp.	60	*Apterostigma dentigerum*	4	EU283929.1	*ActAcro1*	98.02	6×10^{-125}
Pseudonocardia sp.	27	*Acromyrmex octospinosus*	4	EU928982.1	*ActAcro1*	100	7×10^{-134}
Pseudonocardia sp.	55	*Apterostigma dentigerum*	4	EU928990.1	*ActAcro1*	100	7×10^{-134}
Pseudonocardia sp.	52	*Apterostigma dentigerum*	4	EU928993.1	*ActAcro1*	99.61	3×10^{-132}
Pseudonocardia sp.	56	*Apterostigma dentigerum*	4	EU928995.1	*ActAcro1*	100	7×10^{-134}
Pseudonocardia sp.	57	*Apterostigma dentigerum*	4	EU928996.1	*ActAcro1*	100	7×10^{-134}
Pseudonocardia sp.	58	*Apterostigma dentigerum*	4	EU928997.1	*ActAcro1*	100	7×10^{-134}
Pseudonocardia sp.	44	*Apterostigma sp.*	5	EU928989.1	*ActAcro1*	100	6×10^{-134}
Pseudonocardia sp.	54	*Apterostigma dentigerum*	5	EU928994.1	*ActAcro1*	100	6×10^{-134}
Pseudonocardia sp.	100	*Paratrachymyrmex cornetzi*	5	EU929018.1	*ActAcro1*	100	7×10^{-134}
Pseudonocardia sp.	101	*Paratrachymyrmex cornetzi*	5	EU929019.1	*ActAcro1*	100	7×10^{-134}
Pseudonocardia sp.	102	*Paratrachymyrmex cornetzi*	5	EU929020.1	*ActAcro1*	100	7×10^{-134}
Pseudonocardia sp.	103	*Paratrachymyrmex cornetzi*	5	EU929021.1	*ActAcro1*	100	7×10^{-134}
Pseudonocardia sp.	9	*Acromyrmex echinatior*	6	EF588206.2	*ActAcro1*	100	5×10^{-134}
Pseudonocardia sp.	67	*Atta colombica*	6	EF588207.1	*ActAcro1*	100	5×10^{-134}
Pseudonocardia sp.	8	*Acromyrmex echinatior*	6	EF588209.1	*ActAcro1*	100	7×10^{-134}
Pseudonocardia sp.	22	*Acromyrmex octospinosus*	6	EF588210.1	*ActAcro1*	100	7×10^{-134}
Pseudonocardia sp.	105	*Paratrachymyrmex cornetzi*	6	EF588216.1	*ActAcro1*	99.21	2×10^{-130}
Pseudonocardia sp.	109	*Mycetomoellerius zeteki*	6	EF588229.1	*ActAcro1*	100	5×10^{-134}
Pseudonocardia sp.	98	*Paratrachymyrmex cornetzi*	6	EU139572.2	*ActAcro1*	100	5×10^{-134}
Pseudonocardia sp.	2	*Acromyrmex echinatior*	6	EU139574.2	*ActAcro1*	100	7×10^{-134}
Pseudonocardia sp.	3	*Acromyrmex echinatior*	6	EU139580.2	*ActAcro1*	100	5×10^{-134}
Pseudonocardia sp.	4	*Acromyrmex echinatior*	6	EU139581.2	*ActAcro1*	99.61	2×10^{-132}
Pseudonocardia sp.	5	*Acromyrmex echinatior*	6	EU139582.2	*ActAcro1*	100	5×10^{-134}
Pseudonocardia sp.	19	*Acromyrmex octospinosus*	6	EU139584.2	*ActAcro1*	100	5×10^{-134}
Pseudonocardia sp.	106	*Paratrachymyrmex cornetzi*	6	EU283920.1	*ActAcro1*	99.21	2×10^{-130}
Pseudonocardia sp.	32	*Acromyrmex octospinosus*	6	EU283936.1	*ActAcro1*	100	7×10^{-134}
Pseudonocardia sp.	25	*Acromyrmex octospinosus*	6	EU928975.1	*ActAcro1*	100	7×10^{-134}
Pseudonocardia sp.	18	*Acromyrmex octospinosus*	6	EU928976.1	*ActAcro1*	100	6×10^{-134}
Pseudonocardia sp.	17	*Acromyrmex octospinosus*	6	EU928977.1	*ActAcro1*	100	7×10^{-134}
Pseudonocardia sp.	20	*Acromyrmex octospinosus*	6	EU928978.1	*ActAcro1*	100	7×10^{-134}
Pseudonocardia sp.	23	*Acromyrmex octospinosus*	6	EU928980.1	*ActAcro1*	100	7×10^{-134}
Pseudonocardia sp.	26	*Acromyrmex octospinosus*	6	EU928981.1	*ActAcro1*	100	7×10^{-134}
Pseudonocardia sp.	28	*Acromyrmex octospinosus*	6	EU928983.1	*ActAcro1*	100	7×10^{-134}
Pseudonocardia sp.	68	*Atta colombica*	6	EU929003.1	*ActAcro1*	100	4×10^{-134}
Pseudonocardia sp.	125	*Mycetomoellerius zeteki*	?	EU283918.2	*ActAcro1*	97.24	2×10^{-122}
Pseudonocardia sp.	59	*Apterostigma dentigerum*	?	EU928992.1	*ActAcro1*	100	7×10^{-134}

Table S9: *Continued*

Pseudonocardia sp.	95	Myrmicocrypta sp	[2]	EU929015.1	Actin14	99.61	3×10^{-132}
Pseudonocardia sp.	92	Mycocepurus smithii	[2]	EU929016.1	Actin14	99.21	2×10^{-130}
Pseudonocardia sp.	10	Acromyrmex echinatior	[4]	EU928973.1	ActAcro1	100	7×10^{-134}
Pseudonocardia sp.	21	Acromyrmex octospinosus	[4]	EU928979.1	ActAcro1	100	7×10^{-134}
Pseudonocardia sp.	36	Acromyrmex sp	[4]	EU928987.1	ActAcro1	100	5×10^{-134}
Actinobispora aurantiaca	n/a			AF056707.1	ActAcro1	96.88	1×10^{-121}
Actinobispora alaniniphila	n/a			AF056708.1	ActAcro1	94.92	8×10^{-114}
Actinobispora xinjiangensis	n/a			AF056709.1	ActAcro1	95.31	1×10^{-116}
Pseudonocardia zijingensis	n/a			AF325725.1	ActAcro1	98.82	3×10^{-128}
Actinobispora alaniniphila	n/a			AF325726.1	ActAcro1	98.81	3×10^{-128}
Actinobispora aurantiaca	n/a			AF325727.1	ActAcro1	99.21	5×10^{-130}
Actinobispora xinjiangensis	n/a			AF325728.1	ActAcro1	98.81	9×10^{-128}
Pseudonocardia sulfidoxydans	n/a			AF378364.1	ActAcro1	96.06	3×10^{-117}
Pseudonocardia chlorethenivorans	n/a			AF454510.1	ActAcro1	98.02	6×10^{-125}
Pseudonocardia tetrahydrofuranoxydans	n/a			AJ249200.1	ActAcro1	96.05	1×10^{-116}
Pseudonocardia spinospora	n/a			AJ249206.1	ActAcro1	98.03	2×10^{-125}
Pseudonocardia hydrocarbonoxydans	n/a			AJ252826.1	ActAcro1	96.05	1×10^{-116}
Pseudonocardia petroleophila	n/a			AJ252828.1	ActAcro1	97.63	3×10^{-123}
Soil bacterium strain LM 157	n/a			AJ252833.1	ActAcro1	100	7×10^{-134}
Pseudonocardia benzenivorans	n/a			AJ556156.1	ActAcro1	96.08	4×10^{-117}
Pseudonocardia antarctica	n/a			AJ576010.1	ActAcro1	98.8	1×10^{-126}
Pseudonocardia dioxanivorans	n/a			AY340622.1	ActAcro1	96.06	3×10^{-117}
Pseudonocardia sp.	n/a			AY376889.1	ActAcro1	100	6×10^{-134}
Pseudonocardia sp.	n/a			AY376890.1	ActAcro1	100	6×10^{-134}
Pseudonocardia ammonioxydans strain H9	n/a			AY500143.1	ActAcro1	100	7×10^{-134}
Pseudonocardia yunnanensis	n/a			D85472.1	ActAcro1	98.81	3×10^{-128}
Pseudonocardia oroxyli strain D10	n/a			DQ343154.2	ActAcro1	98.03	2×10^{-125}
Pseudonocardia endophytica strain YIM 56035	n/a			DQ887489.2	ActAcro1	99.61	3×10^{-132}
Pseudonocardia sp.	n/a			EF588208.1	ActAcro1	100	7×10^{-134}
Pseudonocardia sp.	n/a			EF588212.1	ActAcro1	100	7×10^{-134}
Pseudonocardia sp.	n/a			EF588214.1	ActAcro1	100	7×10^{-134}
Pseudonocardia saturnea strain NRRL B-16172	n/a			EF588217.2	ActAcro1	98.81	2×10^{-128}
Pseudonocardia thermophila strain NRRL B-1978	n/a			EF588218.2	Actin14	98.43	3×10^{-127}
Pseudonocardia sp.	n/a			EF588219.1	ActAcro1	100	7×10^{-134}
Pseudonocardia sp.	n/a			EF588221.1	ActAcro1	100	7×10^{-134}
Pseudonocardia sp.	n/a			EF588222.1	ActAcro1	100	7×10^{-134}
Streptomyces griseus strain NRRL B-2165	n/a			EF588223.2	12040	93.7	1×10^{-106}
Pseudonocardia sp.	n/a			EF588225.2	ActAcro1	100	7×10^{-134}
Pseudonocardia sp.	n/a			EF588231.2	ActAcro1	100	5×10^{-134}
Pseudonocardia halophobica	n/a			EU139576.2	ActAcro1	98.43	3×10^{-127}
Pseudonocardia sp.	n/a			EU139577.2	ActAcro1	99.21	1×10^{-130}
Pseudonocardia sp.	n/a			EU139578.2	ActAcro1	100	5×10^{-134}
Pseudonocardia sp.	n/a			EU139579.2	ActAcro1	100	5×10^{-134}
Pseudonocardia fici strain YIM 56250	n/a			EU200678.1	ActAcro1	99.61	3×10^{-132}
Pseudonocardia callicarpae strain YIM 56051	n/a			EU200680.1	ActAcro1	99.61	3×10^{-132}
Pseudonocardia sp.	n/a			EU283923.1	ActAcro1	98.02	6×10^{-125}
Pseudonocardia sp.	n/a			EU283924.1	Actin14	99.21	2×10^{-130}
Pseudonocardia sp.	n/a			EU283927.1	ActAcro1	99.61	3×10^{-132}
Pseudonocardia sp.	n/a			EU283931.1	ActAcro1	99.21	1×10^{-130}
Pseudonocardia sp.	n/a			EU283932.1	ActAcro1	100	7×10^{-134}
Pseudonocardia sp.	n/a			EU283935.1	ActAcro1	98.02	5×10^{-125}
Pseudonocardia alaniniphila strain YIM 16303	n/a			EU722519.1	ActAcro1	98.81	3×10^{-128}
Pseudonocardia xinjiangensis strain XJ 45	n/a			EU722520.1	ActAcro1	99.21	6×10^{-130}
Streptomyces sampsonii	n/a			EU928970.1	12040	94.07	3×10^{-108}
Pseudonocardia compacta	n/a			EU928971.1	ActAcro1	98.04	1×10^{-125}
Pseudonocardia autotrophica	n/a			EU928972.1	ActAcro1	99.21	1×10^{-130}
Pseudonocardia sp.	n/a			EU928974.1	ActAcro1	99.61	3×10^{-132}
Pseudonocardia sp.	n/a	Acromyrmex sp.		EU928984.1	ActAcro1	100	6×10^{-134}
Pseudonocardia sp.	n/a	Acromyrmex sp.		EU928985.1	ActAcro1	100	7×10^{-134}
Pseudonocardia sp.	n/a	Acromyrmex sp.		EU928986.1	ActAcro1	100	4×10^{-134}
Pseudonocardia sp.	n/a	Acromyrmex sp.		EU928988.1	ActAcro1	100	7×10^{-134}
Pseudonocardia sp.	n/a	Apterostigma sp.		EU928998.1	ActAcro1	99.61	3×10^{-132}
Pseudonocardia sp.	n/a	Apterostigma sp.		EU928999.1	ActAcro1	100	7×10^{-134}
Pseudonocardia sp.	n/a	Apterostigma sp.		EU929000.1	ActAcro1	100	7×10^{-134}
Pseudonocardia sp.	n/a	Apterostigma sp.		EU929001.1	ActAcro1	100	6×10^{-134}
Pseudonocardia sp.	n/a	Apterostigma sp.		EU929002.1	ActAcro1	100	6×10^{-134}
Pseudonocardia sp.	n/a	Mycetarotes parallelus		EU929007.1	ActAcro1	98.02	6×10^{-125}
Pseudonocardia sp.	n/a	Mycetarotes parallelus		EU929008.1	ActAcro1	97.63	3×10^{-123}
Pseudonocardia sp.	n/a	Mycetarotes parallelus		EU929009.1	ActAcro1	97.63	3×10^{-123}
Pseudonocardia sp.	n/a	Mycetarotes parallelus		EU929010.1	ActAcro1	98.02	6×10^{-125}
Pseudonocardia sp.	n/a	Mycetarotes parallelus		EU929011.1	ActAcro1	98.02	6×10^{-125}
Pseudonocardia sp.	n/a	Mycetarotes parallelus		EU929012.1	ActAcro1	98.02	6×10^{-125}
Pseudonocardia sp.	n/a	Mycetarotes parallelus		EU929013.1	ActAcro1	98.02	6×10^{-125}
Pseudonocardia sp.	n/a	Mycetarotes parallelus		EU929014.1	ActAcro1	98.02	6×10^{-125}
Pseudonocardia alni	n/a			GU083568.1	ActAcro1	100	7×10^{-134}
Pseudonocardia hydrocarbonoxydans	n/a			GU083569.1	ActAcro1	98.02	5×10^{-125}
Pseudonocardia petroleophila	n/a			GU083570.1	ActAcro1	98.02	6×10^{-125}
Amycolata petrophilea	n/a			X55608.1	ActAcro1	96.05	2×10^{-119}
Amycolata nitrificans	n/a			X55609.1	ActAcro1	98.82	5×10^{-130}
Pseudonocardia asaccharolytica	n/a			Y08536.1	ActAcro1		8×10^{-119}
Pseudonocardia sulfidoxydans	n/a			Y08537.1	ActAcro1	95.67	2×10^{-115}

Table S10. Heatmap showing percentage identities of the most abundant unique *Pseudonocardia* sequences within the *Pseudonocardia* OTU *ActAcro1*, identified in the gut microbiomes and cuticular microbiomes of Panamanian fungus-growing ants. The heatmap is based on a distance matrix of the sequence similarities (percentage identity) produced by aligning the most common and abundant unique sequences identified in this study (Cuticle 1-5) and in a previous study (gut and surrounding organs (Gut 1-4); (7)); the intensity of red coloration increases with higher similarities. Labels towards the top and left show the sample origins (gut or cuticular chest plates) and labels towards the right show the single or multiple attine ant species for which the sequences could be obtained, and the proportional abundances of reads for each unique sequence out of the total number of reads for *Pseudonocardia* across all samples in the respective datasets – either the gut (for unique sequences Gut1 – Gut4) or cuticular microbiome (for unique sequences Cuticle1 – Cuticle5). The "*Ps1-Ps2*" sequence is a representative sequence, i.e. the inferred sequence with the smallest total distance to all other sequences in the OTU) generated from amidst the known *Acromyrmex*–associated *Pseudonocardia* strains, which for this sequence region is also known to be identical between the two major cuticular *Pseudonocardia* species *Ps1* (*P. octospinosus*) and *Ps2* (*P. echinatior*) (3, 9, 10). This comparison shows that, for sequences within the *ActAcro1* OTU, the most abundant unique sequence variant that can be retrieved across gut samples (Gut 2) is highly similar but not 100% identical to the most abundant unique sequence variant across the cuticular samples obtained in the present study (Cuticle 5).

	**Unique sequences isolated from attine ant gut and cuticle microbiomes**									**Host species from which unique sequences were isolated**	**Total abundances (%) of each unique sequence across all individuals sampled, for the gut or cuticular microbiome**
	**Gut 1**	**Gut 2**	**Gut 3**	**Gut 4**	**Cuticle 1**	**Cuticle 2**	**Cuticle 3**	**Cuticle 4**	**Cuticle 5**		
**Gut 1**										*Cc*	0.04
**Gut 2**	98.8									*Ms, Cc, Mz, Ac, Ace*	80.64
**Gut 3**	97.6	98.8								*Ms*	8.08
**Gut 4**	98	99.2	99.6							*Ad, Cr, Sa, Mz, Pc, Ae, Ao, Ac, Ace, As*	10.10
**Cuticle 1**	97.6	98.8	99.2	99.6						*Ae*	2.67
**Cuticle 2**	97.6	98.8	99.2	99.6	99.2					*Ae*	1.09
**Cuticle 3**	97.6	98.8	99.2	99.6	99.2	99.2				*Ad*	0.20
**Cuticle 4**	97.6	98.8	99.2	99.6	99.2	99.2	99.2			*Ad*	0.08
**Cuticle 5**	98	99.2	99.6	100	99.6	99.6	99.6	99.6		*Ae, Ao, Pc, Mz, Sa, Cc, Ad, Me, Ms*	95.65
**"Ps1-Ps2"**	98	99.2	99.6	100	99.6	99.6	99.6	99.6	100	*Ae, Ao*	

* Attine species codes: Ace = *Atta cephalotes*; Ac = *Atta colombica*; As = *Atta sexdens*; Ae = *Acromyrmex echinatior*; Ao = *Acromyrmex octospinosus*; Pc = *Paratrachymyrmex cornetzi*; Mz = *Mycetomoellerius zeteki*; Sa = *Sericomyrmex amabilis*; Cc = *Cyphomyrmex costatus*; Cr = *Cyphomyrmex rimosus*; Ad = *Apterostigma dentigerum*; Ms = *Mycocepurus smithii*; Me = *Myrmicocrypta ednaella*

Table S11: Output from DESEQ analyses showing the gut bacterial OTUs (from (7)) that are differentially abundant in host ant species where the *ActAcro1* OTU has been found in the cuticular microbiome, relative to other host ant species where *ActAcro1* was absent in the cuticular microbiome (this study). These results complement earlier analyses by Sapountzis et al. (7) that considered the differential abundance of OTUs in the gut microbiome based the reported presence/absence of an unspecified white 'bloom' of Actinobacteria on the cuticle of ant workers. A positive effect size means that OTUs are more abundant on those ants with *ActAcro1* present and a negative effect size that OTUs are more abundant on those ants with *ActAcro1* absent. While we never found *Amycolatopsis* in cuticular microbiomes of field workers, the present analysis shows that this actinobacterial genus is occasionally represented in the gasters of attine ants that lack *ActAcro1*strains such as *Ps1* and *Ps2* on the cuticle.

OTU Taxonomy	Mean difference	Effect size	SE	z	P
Entomoplasma (unclassified)	260.71	12.72	2.33	5.47	<0.0001
Wolbachia	1800.52	11.92	0.78	15.36	<0.0001
Comamonadaceae (unclassified)	8.94	-2.94	0.82	-3.61	0.00031
Pseudomonas (unclassified)	5.92	-3.45	0.98	-3.53	0.00042
Mycobacterium	6.89	-5.43	1.45	-3.75	0.00018
Labrys (unclassified)	8.53	-6.85	1.90	-3.60	0.00031
Other *Pseudonocardia*	10.86	-7.41	1.81	-4.09	0.00004
Bradyrhizobium (unclassified)	13.47	-8.16	1.88	-4.34	0.00001
Amycolatopsis	29.55	-9.43	1.68	-5.61	<0.0001
Mesorhizobium (unclassified)	278.39	-10.46	1.56	-6.69	<0.0001
Spiroplasma (unclassified)	1316.29	-14.54	1.94	-7.50	<0.0001
Conexibacter (unclassified)	5.12	-24.51	2.86	-8.58	<0.0001
Niabella (unclassified)	13.45	-26.45	1.97	-13.42	<0.0001
Chitinophaga (unclassified)	18.30	-26.91	2.33	-11.53	<0.0001

Table S12: Overview table of our literature survey of all previous studies (pre-2018) that reported associations between *Pseudonocardia* and workers of attine ants, including detailed explanations of our four categories of increasing stringency for accepting reported association as representing natural (i.e. field) associations. These categories produced the data that we used in the columns of Figure 4, which from left to right refer to “confirmations” based on: (i) Any inferred association of *Pseudonocardia* with attine workers sampled in North/Central/South America, through observation of visible blooms on cuticular patches or morphological identification of actinobacterial filamentous growth, either based on light microscopy, or on isolation and DNA-sequencing bacteria collectively or after culturing strains on agar plates, where whole ant washes, macerations or sampling of specific tissues were used for inoculation. (ii) The subset of studies from column (i) that used specific methods to identify cuticular bacteria (e.g. external washes or dissection of chest plates and subsequent identification of *Pseudonocardia* (if present) via DNA sequencing), while excluding studies based on identification by mere observation of bloom alone and those where whole ants (individually or pooled) were macerated. (iii) The subset of studies from column (ii) that sampled multiple workers from more than a single colony, and/or isolated *Pseudonocardia* from multiple colonies of attine ants collected in Panama (the same field site as used in the present study and in (7)). (iv) The subset of studies listed in column (iii) that explicitly identified one or both of the known *Acromyrmex*-associated species, *P. octospinosus* (*Ps1*) and *P. echinatior* (*Ps2*), as originally identified by (9) and (3), and described as proper bacterial species by (10).

Available as a separate file: Table S12.xlsx

4 R scripts

4.1 Generalized Linear Mixed Models

```
library(glmmTMB)
library(car)
library(emmeans)
library(multcomp)


# Load data files. NB these are derived from tables S1a and S1b, but have had spaces replaced by
underscores
actin = read.csv(TablesS1a.csv) #Rarefied dataset
actin2 = read.csv(TablesS1b.csv) #Unrarefied dataset

##Richness analysis (Table S2)

#Overall richness
fitNB1 <- glmmTMB(A_OTU_richness ~ species*Worker_age + (1 | Colony_ID), family = nbinom2, data =
actin)
summary(fitNB1)
Anova(fitNB1)
marginal = emmeans(fitNB1, ~ species)
cld(marginal, alpha=0.05, Letters=letters, adjust="sidak")
# estimate random effect p by treating as a main effect
fitNB1a <- glmmTMB(Total_OTU_richness ~ species*Worker_age, family = nbinom2, data = actin)
anova(fitNB1,fitNB1a)

#Test origin and visible bloom effects
fitNB1c <- glmmTMB(Total_OTU_richness ~ Visible_bacterial_bloom. + (1 | species/Colony_ID), family =
nbinom2, data = actin)
fitNB1d <- glmmTMB(Total_OTU_richness ~ Origin + (1 | species/Colony_ID), family = nbinom2, data =
actin)
fitNB1c
Anova(fitNB1c)
fitNB1d
Anova(fitNB1d)

#Then repeated with dataset actin replaced by dataset actin 2

#Actinobacterial richness
fitNB1 <- glmmTMB(A_OTU_richness ~ species*Worker_age + (1 | Colony_ID), family = nbinom2, data =
actin)
summary(fitNB1)
Anova(fitNB1)
marginal = emmeans(fitNB1, ~ species)
cld(marginal, alpha=0.05, Letters=letters, adjust="sidak")
# get random effect p
fitNB1a <- glmmTMB(A_OTU_richness ~ species*Worker_age, family = nbinom2, data = actin)
anova(fitNB1,fitNB1a)

#test origin and visible bloom
fitNB1c <- glmmTMB(A_OTU_richness ~ Visible_bacterial_bloom. + (1 | species/Colony_ID), family =
nbinom2, data = actin)
fitNB1d <- glmmTMB(A_OTU_richness ~ Origin + (1 | species/Colony_ID), family = nbinom2, data = actin)
fitNB1c
Anova(fitNB1c)
fitNB1d
Anova(fitNB1d)

#Then repeated with dataset actin replaced by dataset actin2
```

```
##Abundance analysis (Table S4)

#Actinobacterial abundance (rarefied dataset)

fit3a <- glmmTMB(Sum_of_A_OTUs ~ species + Worker_age + (1 | Colony_ID), family = nbinom2, data =
actin)
summary(fit3a)
Anova(fit3a)
marginal4 = emmeans(fit3a, ~ species)
cld(marginal4, alpha=0.05, Letters=letters, adjust="Sidak")
# get random effect p
fit3aa <- glmmTMB(Sum_of_A_OTUs ~ species + Worker_age, family = nbinom2, data = actin)
anova(fit3aa,fit3a)
#test origin and visible bloom
fit3c <- glmmTMB(Sum_of_A_OTUs ~ Visible_bacterial_bloom. + (1 | species/Colony_ID), family =
nbinom2, data = actin)
fit3d <- glmmTMB(Sum_of_A_OTUs ~ Origin + (1 | species/Colony_ID), family = nbinom2, data = actin)
fit3c
Anova(fit3c)
fit3d
Anova(fit3d)

#Pseudonocardia abundance (unrarefied dataset)

fit3a <- glmmTMB(Sum_of_P_OTUs ~ species + Worker_age + (1 | Colony_ID), family = nbinom2, data =
actin2)
summary(fit3a)
Anova(fit3a)
marginal4 = emmeans(fit3a, ~ species)
cld(marginal4, alpha=0.05, Letters=letters, adjust="Sidak")
# get random effect p
fit3aa <- glmmTMB(Sum_of_P_OTUs ~ species + Worker_age, family = nbinom2, data = actin2)
anova(fit3aa,fit3a)
#test origin and visible bloom
fit3c <- glmmTMB(Sum_of_P_OTUs ~ Visible_bacterial_bloom. + (1 | species/Colony_ID), family =
nbinom2, data = actin2)
fit3d <- glmmTMB(Sum_of_P_OTUs ~ Origin + (1 | species/Colony_ID), family = nbinom2, data = actin2)
fit3c
Anova(fit3c)
fit3d
Anova(fit3d)
```

```
###Prevalence analysis (Table S5)

#Actinobacteria out of All
fitB1 <- glmmTMB(cbind(Sum_of_A_OTUs, Sum_of_All_OTUs) ~ species*Worker_age + (1 | Colony_ID), family
= betabinomial, data = actin)
summary(fitB1)
Anova(fitB1)
marginal = emmeans(fitB1, ~ species)
cld(marginal, alpha=0.05, Letters=letters, adjust="sidak")
# get random effect p
fitB1a <- glmmTMB(cbind(Sum_of_A_OTUs, Sum_of_All_OTUs) ~ species*Worker_age, family = betabinomial,
data = actin)
anova(fitB1,fitB1a)
#test origin and visible bloom effects
fitB1c <- glmmTMB(cbind(Sum_of_A_OTUs, Sum_of_All_OTUs) ~ Visible_bacterial_bloom. + (1 | Colony_ID),
family = betabinomial, data = actin)
fitB1d <- glmmTMB(cbind(Sum_of_A_OTUs, Sum_of_All_OTUs) ~ Origin + (1 | Colony_ID), family =
betabinomial, data = actin)
fitB1c
Anova(fitB1c)
fitB1d
Anova(fitB1d)

#Then repeated with dataset actin replaced by dataset actin2

#Pseudonocardia out of Actinobacteria

fitP1 <- glmmTMB(cbind(Sum_of_P_OTUs, Sum_of_A_OTUs) ~ species+Worker_age + (1 | Colony_ID), family =
betabinomial, data = actin)
summary(fitP1)
Anova(fitP1)
marginalP = emmeans(fitP1, ~ species)
cld(marginalP, alpha=0.05, Letters=letters, adjust="sidak")

# get random effect p
fitP1a <- glmmTMB(cbind(Sum_of_P_OTUs, Sum_of_A_OTUs) ~ species+Worker_age, family = betabinomial,
data = actin)
anova(fitP1,fitP1a)

#get origin and visible bloom effects
fitP1c <- glmmTMB(cbind(Sum_of_P_OTUs, Sum_of_A_OTUs) ~ Visible_bacterial_bloom. + (1 | Colony_ID),
family = betabinomial, data = actin)
fitP1d <- glmmTMB(cbind(Sum_of_P_OTUs, Sum_of_A_OTUs) ~ Origin + (1 | Colony_ID), family =
betabinomial, data = actin)
fitP1c
Anova(fitP1c)
fitP1d
Anova(fitP1d)

#Then repeated with dataset actin replaced by dataset actin2
```

DESeq 2 analysis

```
countTable<-read.table("unrarefied", header=T, row.names = 2)
groups<-countTable[1:11]
rest<-countTable[-c(1:11)]
Design1<-groups[,10]
Design1<-as.factor(Design1)
otu=import_mothur(mothur_shared_file = "unrarefied_OTUtable.txt",
mothur_constaxonomy_file="unrarefied.taxonomy", cutoff=0.03)
tax_table(otu.Pseudo)
#subset based on taxonomy
otu.Actino = subset_taxa(otu, Rank2 == "Actinobacteria")
otu.Pseudo = subset_taxa(otu, Rank6 == "Pseudonocardineae")

#sum pseudo abundances and make a new column showing whether there is a presence or not
new<-otu_table(otu.Pseudo)
new<-t(new)
R.Pseudo=subset(rest, select=colnames(new))
groups$Pseudo.sum=rowSums(R.Pseudo)
groups$Pseudo.presence = ifelse(groups$Pseudo.sum > 1, "yes", "no")

new<-otu_table(otu.Actino)
new<-t(new)
R.Actino=subset(rest, select=colnames(new))
groups$Actino.sum=rowSums(R.Actino)
groups$Actino.presence = ifelse(groups$Actino.sum > 1, "yes", "no")

plot(groups$Actino.sum, groups$Pseudo.sum)
text(groups$Actino.sum, groups$Pseudo.sum, labels=groups$species, cex= 0.7, pos=3)

Design1<-groups[,15]

#remove samples that have 0
otu.Actino2 = prune_samples(sample_sums(otu.Actino)>=1, otu.Actino)
groups=t(groups)
groups2<-subset(groups, select=sample.names(otu.Actino2))
groups2=t(groups2)
Design1<-groups2[,15]

groups=as.data.frame(groups)
plot(groups$species, groups$Pseudo.sum)

fit1 <- glm.nb(Actino.sum ~ species, data=groups)
anova(fit)
fit2 <- zeroinfl(Actino.sum ~  species|1, dist = c("negbin"), data=groups)

summary(fit1)
anova(fit1)
AIC(fit1,fit2)
vuong(fit1,fit2)

contrasts <- contrast(lsmeans(fit1, list(~species)), adjust="tukey")
```

5 Supplementary References

1. C. R. Currie, J. A. Scott, R. C. Summerbell, D. Malloch, Fungus-growing ants use antibiotic-producing bacteria to control garden parasites. *Nature* **398**, 701 (1999).
2. C. R. Currie, M. Poulsen, J. Mendenhall, J. J. Boomsma, J. Billen, Coevolved crypts and exocrine glands support mutualistic bacteria in fungus-growing ants. *Science* **311**, 81-83 (2006).
3. S. B. Andersen, L. H. Hansen, P. Sapountzis, S. J. Sorensen, J. J. Boomsma, Specificity and stability of the *Acromyrmex-Pseudonocardia* symbiosis. *Molecular Ecology* **22**, 4307-4321 (2013).
4. J. G. Caporaso *et al.*, Global patterns of 16S rRNA diversity at a depth of millions of sequences per sample. *Proceedings of the National Academy of Sciences* **108**, 4516-4522 (2011).
5. J. J. Kozich, S. L. Westcott, N. T. Baxter, S. K. Highlander, P. D. Schloss, Development of a dual-index sequencing strategy and curation pipeline for analyzing amplicon sequence data on the MiSeq Illumina sequencing platform. *Applied and Environmental Microbiology* **79**, 5112-5120 (2013).
6. C. Quast *et al.*, The SILVA ribosomal RNA gene database project: improved data processing and web-based tools. *Nucleic Acids Res* **41**, D590-596 (2013).
7. P. Sapountzis, D. R. Nash, M. Schiøtt, J. J. Boomsma, The evolution of abdominal microbiomes in fungus-growing ants. *Molecular Ecology* **28**, 879-899 (2019).
8. Y. Hu *et al.*, By their own devices: invasive Argentine ants have shifted diet without clear aid from symbiotic microbes. *Molecular Ecology* **26**, 1608-1630 (2017).
9. M. Poulsen, M. Cafaro, J. J. Boomsma, C. R. Currie, Specificity of the mutualistic association between actinomycete bacteria and two sympatric species of *Acromyrmex* leaf-cutting ants. *Molecular Ecology* **14**, 3597-3604 (2005).
10. N. A. Holmes *et al.*, Genome analysis of two *Pseudonocardia* phylotypes associated with *Acromyrmex* leafcutter ants reveals their biosynthetic potential. *Frontiers in Microbiology* **7:2073** (2016).
11. I. Scheuring, D. W. Yu, How to assemble a beneficial microbiome in three easy steps. *Ecology Letters* **15**, 1300-1307 (2012).
12. S. F. Worsley *et al.*, Competition-based screening helps to secure the evolutionary stability of a defensive microbiome. *BMC biology* **19**, 1-20 (2021).
13. M. E. Brooks *et al.*, glmmTMB balances speed and flexibility among packages for zero-inflated generalized linear mixed modeling. *The R journal* **9**, 378-400 (2017).
14. R. Lenth, emmeans: Estimated Marginal Means, aka Least-Squares Means. *R package version 1.4.5.* (2020).
15. M. G. Branstetter *et al.*, Dry habitats were crucibles of domestication in the evolution of agriculture in ants. *Proceedings of the Royal Society B: Biological Sciences* **284** (2017).
16. M. I. Love, W. Huber, S. Anders, Moderated estimation of fold change and dispersion for RNA-seq data with DESeq2. *Genome Biology* **15**, 550 (2014).
17. P. Sapountzis *et al.*, *Acromyrmex* leaf-cutting ants have simple gut microbiota with nitrogen-fixing potential. *Applied and Environmental Microbiology* **81**, 5527-5537 (2015).
18. S. F. Altschul, W. Gish, W. Miller, E. W. Myers, D. J. Lipman, Basic local alignment search tool. *Journal of molecular biology* **215**, 403-410 (1990).
19. M. J. Cafaro *et al.*, Specificity in the symbiotic association between fungus-growing ants and protective *Pseudonocardia* bacteria. *Proceedings of the Royal Society B-Biological Sciences* **278**, 1814-1822 (2011).
20. C. R. Currie *et al.*, Ancient tripartite coevolution in the attine ant-microbe symbiosis. *Science* **299**, 386-388 (2003).
21. C. Kost *et al.*, Non-specific association between filamentous bacteria and fungus-growing ants. *Naturwissenschaften* **94**, 821-828 (2007).
22. U. G. Mueller, D. Dash, C. Rabeling, A. Rodrigues, Coevolution between attine ants and actinomycete bacteria: A reevalutaion. *Evolution* **62**, 2894-2912 (2008).

23. S. Haeder, R. Wirth, H. Herz, D. Spiteller, Candicidin-producing *Streptomyces* support leaf-cutting ants to protect their fungus garden against the pathogenic fungus *Escovopsis*. *Proceedings of the National Academy of Sciences* **106**, 4742-4746 (2009).
24. R. Sen *et al.*, Generalized antifungal activity and 454-screening of *Pseudonocardia* and *Amycolatopsis* bacteria in nests of fungus-growing ants. *Proceedings of the National Academy of Sciences of the United States of America* **106**, 17805-17810 (2009).
25. J. Barke *et al.*, A mixed community of actinomycetes produce multiple antibiotics for the fungus farming ant *Acromyrmex octospinosus*. *BMC Biology* **8:109** (2010).
26. S. B. Andersen, S. H. Yek, D. R. Nash, J. J. Boomsma, Interaction specificity between leaf-cutting ants and vertically transmitted *Pseudonocardia* bacteria. *BMC Evolutionary Biology* **15**, 27 (2015).
27. H. Li *et al.*, Convergent evolution of complex structures for ant–bacterial defensive symbiosis in fungus-farming ants. *Proceedings of the National Academy of Sciences* **115**, 10720-10725 (2018).
28. E. A. Barka *et al.*, Taxonomy, physiology, and natural products of Actinobacteria. *Microbiology and Molecular Biology Reviews* **80**, 1-43 (2016).
29. A. M. Maszenan *et al.*, *Granulicoccus phenolivorans* gen. nov., sp. nov., a Gram-positive, phenol-degrading coccus isolated from phenol-degrading aerobic granules. *International Journal of Systematic and Evolutionary Microbiology* **57**, 730-737 (2007).
30. A. B. de Menezes, J. E. McDonald, H. E. Allison, A. J. McCarthy, Importance of *Micromonospora* spp. as colonizers of cellulose in freshwater lakes as demonstrated by quantitative reverse transcriptase PCR of 16S rRNA. *Applied and Environmental Microbiology* **78**, 3495-3499 (2012).
31. D. Lewis *et al.*, The human urinary microbiome; bacterial DNA in voided urine of asymptomatic adults. *Frontiers in Cellular and Infection Microbiology* **3** (2013).
32. C. Noirot, A. Quennedey, Fine structure of insect epidermal glands. *Annual review of entomology* **19**, 61-80 (1974).
33. U. G. Mueller, S. A. Rehner, T. R. Schultz, The Evolution of Agriculture in Ants. *Science* **281**, 2034-2038 (1998).
34. T. R. Schultz, S. G. Brady, Major evolutionary transitions in ant agriculture. *Proceedings of the National Academy of Sciences* **105**, 5435-5440 (2008).
35. P. E. Hanisch, J. Sosa-Calvo, T. R. Schultz, The last piece of the puzzle? phylogenetic position and natural history of the monotypic fungus-farming ant genus *Paramycetophylax* (Formicidae: Attini). *Insect Systematics and Diversity* **6** (2022).
